# Multi-omics Mendelian randomization integrating metabolism, microbiome and immunity supports a putative gut-immune-pelvic pathway in deep infiltrating endometriosis

**DOI:** 10.3389/fendo.2026.1827134

**Published:** 2026-05-21

**Authors:** Shanping Shi, Wei Song, Zhiping Wu, Yufeng Cheng, Hua Liu, Fuju Tian, Xiaocui Li

**Affiliations:** 1 Shanghai Key Laboratory of Maternal Fetal Medicine, Clinical and Translational Research Center of Shanghai First Maternity and Infant Hospital, Shanghai Institute of Maternal-Fetal Medicine and Gynecologic Oncology, Shanghai First Maternity and Infant Hospital, School of Medicine, Tongji University, Shanghai, China; 2 Department of Obstetrics and Gynecology, Ruijin Hospital, Shanghai Jiao Tong University School of Medicine, Shanghai, China

**Keywords:** circulating metabolites, deep infiltrating endometriosis, gut microbiota, immune cell phenotypes, Mendelian randomization, multi-omics

## Abstract

**Background:**

Deep infiltrating endometriosis (DIE) is a highly fibrotic and deeply invasive subtype of endometriosis that causes severe pelvic pain, infertility and marked impairment of quality of life. Metabolic, microbial and immune disturbances have been reported in women with endometriosis, but whether these systemic perturbations causally contribute to DIE and which lesion-level molecular mediators connect them to pelvic pathology remains unknown.

**Methods:**

We performed two-sample Mendelian randomization (MR) to assess the causal effects of circulating metabolites, gut microbiota (GM) traits and immune cell phenotypes on DIE risk using genome-wide association data from FinnGen and large exposure GWAS. Bayesian colocalization was applied to identify protein-coding genes with shared causal variants between exposures and DIE. Colocalized genes were integrated with RNA-sequencing data from GSE141549 (normal endometrium, n = 43; DIE lesions, n = 88) to evaluate differential expression and immune-cell associations inferred by CIBERSORT-like deconvolution. Machine-learning-based feature selection was used to derive a multigene logistic model, and protein expression of feature genes was validated by immunohistochemistry in independent specimens.

**Results:**

MR revealed putative causal associations between multiple circulating metabolites, GM taxa and immune phenotypes and DIE susceptibility, including risk-increasing bile acid-related and acylcarnitine species, specific bacterial taxa, and monocytic/dendritic-cell traits, and protective lipid species, short-chain-fatty-acid-linked genera and CD45RA^-^CD4^+^ T-cell subsets. Colocalization identified 324 protein-coding genes, of which 42 were differentially expressed between DIE and controls and enriched in inflammatory and extracellular matrix remodeling pathways. A five-gene panel—HDC, GADD45B, CDK5, AHNAK and RASGRP2—was prioritized, showed structured correlations with B-cell, NK-cell and CD4^+^ memory T-cell subsets, and showed excellent within-cohort discrimination between DIE lesions and normal endometrium (AUC = 0.999). Immunohistochemistry confirmed upregulation of HDC, GADD45B, AHNAK and RASGRP2 and downregulation of CDK5 in DIE lesions.

**Conclusion:**

This multi-omics MR framework supports a putative gut-immune-pelvic pathway in DIE and identifies a biologically plausible five-gene tissue-level signature consistent with lesion-associated fibrotic and immune-inflammatory remodeling.

## 1 Introduction

Endometriosis is a chronic inflammatory gynecologic disorder that affects approximately 10% of reproductive-aged women worldwide and is a major cause of pelvic pain, infertility, and impaired quality of life (1). Deep infiltrating endometriosis (DIE) represents the most aggressive phenotype, defined by endometriotic nodules penetrating >5 mm beneath the peritoneal surface and frequently involving the rectovaginal septum, uterosacral ligaments, bowel, and bladder; it is strongly associated with severe dysmenorrhea, deep dyspareunia, dyschezia, and marked reductions in health-related quality of life (2). Despite advances in surgical techniques and hormonal therapies, the etiology of DIE remains incompletely understood and likely reflects complex interactions between genetic susceptibility and systemic as well as local environmental factors (3).

Metabolic and immunological dysregulation are established features of the endometriosis microenvironment, and accumulating data also implicate gut microbiota disturbances as an additional layer of disease-associated perturbation. Targeted and untargeted metabolomics consistently show disturbances in amino-acid, lipid and organic-acid metabolism in serum and peritoneal fluid from women with moderate-to-severe endometriosis, with altered glycolytic and tricarboxylic-acid-cycle intermediates and acylcarnitine and phospholipid species indicative of increased energy demand, mitochondrial stress and oxidative imbalance (4). Evidence synthesized from human and experimental studies further indicates that endometriosis is accompanied by gut microbiota dysbiosis, characterized by reduced microbial diversity, shifts in the Firmicutes-to-Bacteroidetes ratio and enrichment of taxa such as Prevotella and Escherichia coli, changes that may enhance enterohepatic estrogen recycling and mucosal inflammation (5). On the immune side, patients with endometriosis exhibit an accumulation of activated, pro-angiogenic macrophages together with impaired natural killer cell cytotoxicity and expansion of regulatory T cells in the peritoneal cavity, contributing to a tolerogenic, fibrosis-prone niche that favors lesion persistence (3). Collectively, these observations suggest that systemic metabolic traits, gut-derived microbial signals and immune-cell composition jointly shape the initiation and progression of endometriotic lesions, yet their causal relevance for deep infiltrating endometriosis and the molecular mediators linking these exposures to lesion biology remain to be defined.

Mendelian randomization (MR) uses germline genetic variants as instrumental variables for modifiable exposures, providing a powerful framework for causal inference that mitigates confounding and reverse causation inherent to conventional observational studies (6). Large genome-wide association studies have now delineated genetic determinants of circulating metabolites, gut microbiota taxa and immune cell phenotypes, creating an opportunity to systematically assess whether these traits exert causal effects on the risk of deep infiltrating endometriosis (DIE) and to anchor downstream mechanistic investigations in genetically supported pathways (3, 4). However, most work in endometriosis has focused on overall disease susceptibility, isolated exposure domains or single-omics layers, and has rarely combined MR-based causal inference with lesion-level transcriptomic profiling, immune-cell deconvolution and clinically interpretable diagnostic modeling in a DIE-specific setting.

In this study, we used two-sample MR to evaluate the causal effects of genetically proxied circulating metabolites, gut microbiota composition and immune cell phenotypes on the risk of deep infiltrating endometriosis. Bayesian colocalization was then applied to identify protein-coding genes at loci with shared causal variants between exposure traits and DIE. By integrating these genetically prioritized genes with RNA-sequencing data from DIE lesions and control endometrium, we characterized their differential expression, co-expression and immune-infiltration correlates, and used machine-learning–based feature selection to construct and internally evaluate a multigene tissue-level prediction model and nomogram for DIE-related lesion classification. Finally, we assessed protein-level expression of the selected feature genes in independent clinical specimens using immunohistochemistry, providing orthogonal validation of the exposure-related molecular signatures identified by our multi-omics framework.

## 2 Methods

### 2.1 Study design

**Figure 1:**
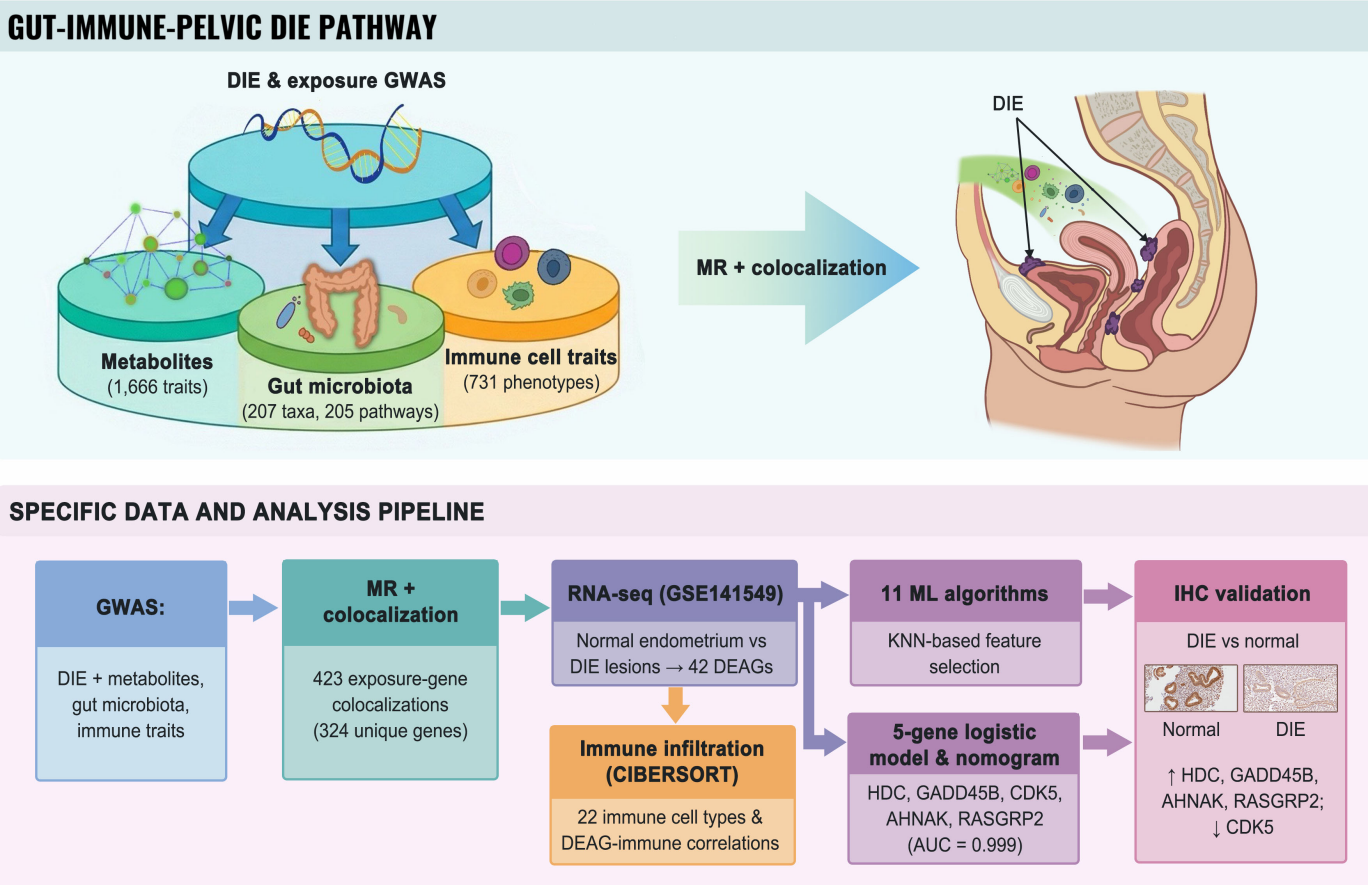
Study overview and analytical workflow. Schematic of a putative gut–immune–pelvic pathway in DIE, deep infiltrating endometriosis, integrating GWAS for circulating metabolites, gut microbiota and immune cell traits with DIE GWAS. The lower panel shows the stepwise pipeline: MR and colocalization, transcriptomic and immune infiltration analyses in GSE141549, machine-learning-based feature selection, construction of a five-gene logistic model and nomogram, and immunohistochemical validation.

We used two-sample Mendelian randomization (MR) to assess the causal effects of circulating metabolites, gut microbiota composition and immune cell populations on the risk of deep infiltrating endometriosis (DIE). Exposures showing significant MR associations with DIE were then subjected to colocalization analysis to identify loci with evidence of shared causal variants. Transcriptomic data from DIE lesions and control endometrium in the GEO database were subsequently analyzed to perform differential expression, correlation and immune infiltration analyses of colocalized genes. Feature genes were identified using machine-learning approaches and incorporated into an internally evaluated tissue-level prediction model, which was assessed in the GEO cohort using receiver operating characteristic (ROC) analysis, calibration and decision curve analysis (DCA), and visualized as a nomogram. Finally, immunohistochemistry was performed on independent DIE and control endometrial specimens to validate protein expression of the selected feature genes (**Figure 1**).

Valid MR inference requires three core assumptions: genetic instruments are strongly associated with the exposure, independent of confounders and influence the outcome only through the exposure. The design, conduct and reporting of our MR analyses followed the STROBE–MR guidelines (7).

### 2.2 Data sources

GWAS summary statistics were obtained from multiple consortia via the Integrative Epidemiology Unit (IEU) OpenGWAS database (https://gwas.mrcieu.ac.uk/). Independent SNPs meeting the predefined significance threshold (P < 5 × 10^-6^) were selected as instrumental variables after linkage disequilibrium clumping (r² < 0.001, 10-Mb windows), and variants with F-statistics ≤ 10 were excluded to reduce weak-instrument bias.

Summary data for DIE were obtained from the FinnGen R12 GWAS of vaginal and rectovaginal septum endometriosis (finn-b-N14_ENDOMETRIOSIS_RECTPVAGSEPT_VAGINA), including 1,360 cases and 68,969 controls of European ancestry (8). Gut microbiota (GM) genetics were derived from the Netherlands Microbiome Project, a shotgun metagenomic GWAS of 7,738 individuals of European ancestry that reported host genetic associations for 207 microbial taxa and 205 microbial metabolic pathways (9). Immune cell genetics were obtained from a Sardinian population-based study of 3,757 participants, encompassing 731 immune phenotypes including cell counts, morphology, surface marker intensities and absolute cell numbers (10). Metabolite genetics were taken from a whole-genome–sequencing-based GWAS of circulating metabolites in up to 11,840 adults, profiling 1,666 plasma metabolites across multiple cohorts (11).

For downstream transcriptomic and modeling analyses, we downloaded GSE141549 from GEO, which contains genome-wide expression profiles of endometrium, peritoneum and multiple endometriosis lesion types from 115 patients and 53 controls (12). In this study, we used normal endometrium from women without endometriosis as controls (n = 43) and deep infiltrating endometriotic lesions as the DIE group (n = 88).

### 2.3 Patients and specimens

In this study, all samples were obtained from women undergoing laparoscopic surgery at Ruijin Hospital, Shanghai Jiao Tong University School of Medicine, Shanghai, China between September and November 2024. Tissue samples of Deep Infiltrating Endometriosis (DIE) (*n* = 5) were collected during the proliferative phase of the menstrual cycle from patients. DIE was defined as endometriotic lesions infiltrating >5 mm beneath the peritoneal surface, and the diagnosis was confirmed by intraoperative findings and histopathology (13). Patients were excluded from tissue collection if they had uterine myomas, adenomyomas, or malignant tumors. Additionally, all participants were free of hormonal medication for at least two menstrual cycles prior to the collection of endometrial samples. Normal endometrial tissues were obtained from women (n = 5) with no pathological abnormalities during hysteroscopy, using an endometrial curette. All participants provided written informed consent, and sample collection was conducted under a protocol approved by the institutional ethics committee of Ruijin Hospital.

### 2.4 Mendelian randomization analysis

Two-sample MR analyses were performed in R (version 4.5.1) using the TwoSampleMR package (version 0.5.6) to evaluate the causal effects of genetically proxied circulating metabolites, GM traits and immune cell populations on DIE (6). Primary analyses used multiplicative random-effects inverse variance–weighted (IVW) models, with MR-Egger, weighted median, weighted mode and MR-PRESSO as complementary sensitivity analyses (14). Heterogeneity across SNP-specific estimates was assessed using Cochran’s Q statistic (P < 0.05 indicating significant heterogeneity), and horizontal pleiotropy was evaluated using the MR-Egger intercept test (P < 0.05 indicating directional pleiotropy). Steiger directionality tests were applied to confirm that instruments explained more variance in the exposure than in DIE, supporting a forward causal direction (15). IVW served as the primary estimator. Domain-specific Bonferroni thresholds were calculated as conservative reference thresholds for multiple testing; however, because the present work was designed as a discovery-oriented integrative screen, associations retained for downstream analyses were additionally required to show IVW P< 0.05, concordant effect directions across sensitivity methods, and no evidence of substantial heterogeneity, directional pleiotropy or major distortion after sensitivity testing.

### 2.5 Colocalization analysis

For exposures showing robust MR associations with DIE, we performed Bayesian colocalization. Gene-centered regions extending ±2500 bp around mapped protein-coding genes were defined, and overlapping SNPs from exposure and DIE GWAS were extracted. Colocalization was conducted using the coloc R package (version 5.2.1), which estimates posterior probabilities for shared versus distinct causal variants based on effect sizes and standard errors from both traits (16). Colocalization was considered supportive when the posterior probability for a shared causal variant (PP.H4) exceeded 0.50; genes at loci with PP.H4 > 0.50 were retained as colocalized candidates for subsequent transcriptomic and functional analyses. Default coloc prior probabilities were used (p1 = 1 × 10^-^4, p2 = 1 × 10^-^4, p12 = 1 × 10^-^5).

### 2.6 Differentially expressed associated gene identification and analysis

Before differential expression analysis, GSE141549 was processed through a standardized workflow. Raw RNA-seq count data were normalized using the DESeq2 package (17), and gene identifiers were mapped to HGNC symbols; genes lacking valid symbols were excluded.

Expression levels of colocalized genes were extracted for differential expression analysis comparing control (n = 43) and DIE (n = 88) samples. Genes with adjusted *P* < 0.05 were defined as differentially expressed associated genes. These genes were mapped to their genomic coordinates and used to construct circos-style plots of chromosomal distribution. Pairwise correlations between differentially expressed associated genes were calculated using Spearman’s rank correlation via the cor() function, and significance was evaluated with cor.test() and Benjamini-Hochberg false discovery rate correction, with *P* < 0.05 considered significant.

### 2.7 Immune cell analysis of DIE samples

Immune cell composition in each sample was inferred using a CIBERSORT-like deconvolution algorithm based on ν-support vector regression with 1,000 permutations to estimate relative proportions of 22 immune cell types (18). Group differences in immune cell fractions between normal endometrium (n = 43) and DIE lesions (n = 88) were assessed using Wilcoxon rank-sum tests implemented in ggpubr and visualized as boxplots and stacked barplots. Correlations between differentially expressed associated genes and immune cell populations were calculated using Spearman’s rank correlation by matching normalized gene expression values with inferred immune cell fractions. *P* < 0.05 was considered statistically significant for group comparisons and gene–immune cell correlations.

### 2.8 Feature gene identification and nomogram development

Expression profiles of differentially expressed associated genes were used to build a panel of machine-learning classifiers to distinguish DIE lesions from normal endometrium. Eleven algorithms—including random forest, support vector machine, generalized linear model, gradient boosting, k-nearest neighbor, neural network, LASSO logistic regression, decision tree, naïve Bayes, AdaBoost and bagging—were implemented in the caret framework using a stratified 70/30 training–test split, with the held-out test set kept fully isolated during feature selection and model fitting. Model comparison was based on 5-fold cross-validated predictive performance in the training set together with residual distributions and ROC curves in the held-out test set. The k-nearest neighbor model provided the most stable and informative feature-importance profile, and its top-ranked predictors were defined as feature genes. A multigene logistic regression model was then fitted with the rms package, and a nomogram was constructed using feature gene expression patterns in control and DIE samples. Discrimination, calibration, and within-cohort decision-analytic performance were assessed in GSE141549 using AUC, bootstrap-corrected calibration curves, the Hosmer−Lemeshow test and DCA (19, 20). These procedures provide an internal evaluation of model behavior within GSE141549 but do not replace external validation.

### 2.9 Immunohistochemical validation of feature genes

Immunohistochemistry was performed on formalin-fixed, paraffin-embedded DIE lesions and normal endometrium to assess protein expression of the five feature genes. After deparaffinization, rehydration and heat-induced antigen retrieval, sections were incubated with primary antibodies against each target, followed by HRP-conjugated secondary antibodies and visualization with 3,3′-diaminobenzidine (DAB) and hematoxylin counterstaining. Digital images were acquired under identical microscope and camera settings. Staining was quantified using ImageJ (NIH): for each section, the integrated optical density (IOD) and corresponding positive-staining area were measured in multiple randomly selected fields, and the mean IOD/area ratio was calculated as an index of relative protein expression for each marker.

### 2.10 Statistical analysis

Statistical analyses were conducted primarily in R (version 4.5.1) using two-sided tests, and immunohistochemical quantification was analyzed in GraphPad Prism. Normality of continuous variables was evaluated with the Shapiro–Wilk test prior to group comparisons. Differences between normal endometrium and DIE lesions were assessed using independent-samples t tests when assumptions of normality and homogeneity of variance were satisfied, and Wilcoxon rank-sum tests otherwise. For IHC, mean IOD/area ratios of each marker were compared between DIE and control tissues using unpaired two-tailed Student’s t-tests. Spearman’s rank correlation coefficients were used to quantify associations between gene expression and immune cell fractions. Multiple testing in differential expression and correlation analyses was controlled using the Benjamini–Hochberg false discovery rate procedure (q < 0.05). For MR analyses, IVW P values were used as the primary screening metric, and Bonferroni thresholds were calculated separately for each exposure domain as conservative reference thresholds for multiple testing. Unless otherwise specified, P < 0.05 was considered statistically significant.

### 2.11 Data availability

GWAS summary statistics for DIE and exposure traits (circulating metabolites, GM traits and immune cell phenotypes) were obtained from the IEU OpenGWAS project and the relevant source consortia and are publicly available through their respective repositories. The transcriptomic dataset used for expression and diagnostic modeling analyses (GSE141549) is available from GEO (12). Clinical IHC data generated in this study are available from the corresponding author upon reasonable request. All publicly available datasets can be accessed through their original databases or via the IEU OpenGWAS platform.

## 3 Results

### 3.1 GWAS and GEO dataset collection

The MR framework integrated GWAS summary statistics for deep infiltrating endometriosis (DIE;1,360 cases and 68,969 controls of European ancestry) with large-scale genetic datasets forcirculating metabolites (1,666 plasma metabolites), gut microbiota (GM; 207 microbial taxa and 205 microbial metabolic pathways) and 731 immune cell phenotypes. All exposure instruments are summarized in **Supplementary Table 1**. For downstream transcriptomic and diagnostic modeling analyses, we used the RNA-sequencing dataset GSE141549, restricting the cohort to normal endometrium from women without endometriosis (n = 43) and DIE lesions (n = 88).

### 3.2 Mendelian randomization analysis

**Figure 2:**
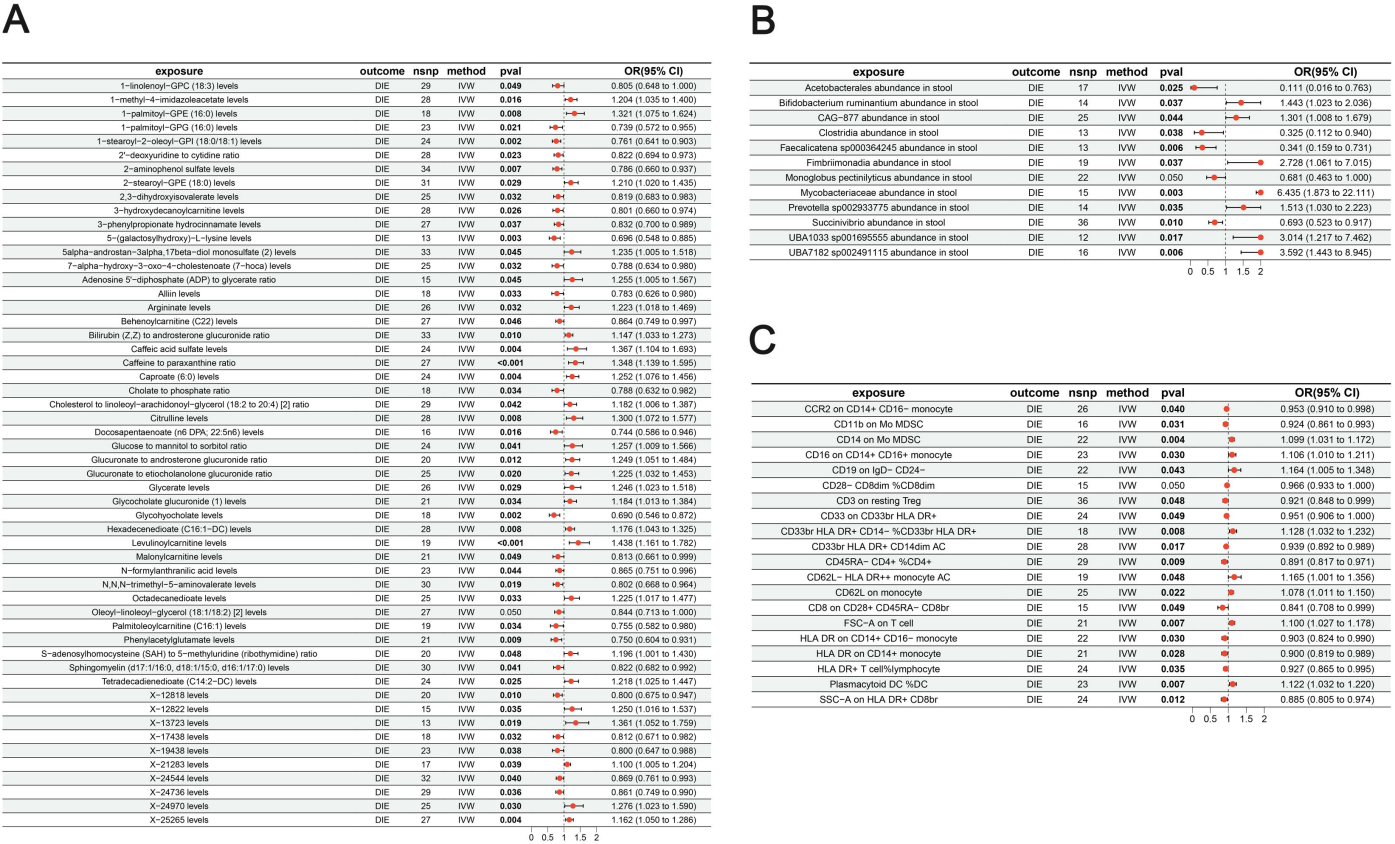
Mendelian randomization associations of multi-omics exposures with DIE. Forest plots of MR estimates for **(A)** circulating metabolites, **(B)** gut microbiota taxa and **(C)** immune cell phenotypes in relation to DIE risk. Odds ratios and 95% confidence intervals are shown for traits meeting predefined MR robustness criteria.

MR identified multiple IVW-significant associations consistent with putative causal effects of exposure traits on DIE risk. Among circulating metabolites, several bile acid–related, acylcarnitine and amino acid metabolites showed notable relationships with disease susceptibility. Higher caffeine to paraxanthine ratio and levulinoylcarnitine levels were associated with increased risk (OR = 1.35, 95% CI = 1.14–1.60, P < 0.001; OR = 1.44, 95% CI = 1.16–1.78, P < 0.001), whereas 1-stearoyl-2-oleoyl-GPI (18:0/18:1), glycohyocholate and 5-(galactosylhydroxy)-L-lysine were associated with lower risk (OR = 0.76, 95% CI = 0.64–0.90, P = 0.002; OR = 0.69, 95% CI = 0.55–0.87, P = 0.002; OR = 0.70, 95% CI = 0.55–0.88, P = 0.003; **Figure 2A**).

GM analysis revealed multiple IVW-significant associations with DIE. Several taxa from distinct bacterial groups showed notable associations, with some acting as risk factors and others as protective traits. For example, higher abundances of Mycobacteriaceae and UBA7182 sp002491115 markedly increased risk (OR = 6.43, 95% CI = 1.87–22.11, P = 0.003; OR = 3.59, 95% CI = 1.44–8.94, P = 0.006), whereas Faecalicatena sp000364245 and Succinivibrio were associated with reduced risk (OR = 0.34, 95% CI = 0.16–0.73, P = 0.006; OR = 0.69, 95% CI = 0.52–0.92, P = 0.010; **Figure 2B**).

Immune cell phenotype analysis likewise suggested multiple IVW-significant associations with DIE. Monocytic, dendritic and T-cell traits showed notable relationships with disease susceptibility. Increased CD14 expression on monocytic myeloid-derived suppressor cells and higher plasmacytoid dendritic cell percentages were associated with elevated risk (OR = 1.10, 95% CI = 1.03–1.17, P = 0.004; OR = 1.12, 95% CI = 1.03–1.22, P = 0.007), whereas higher proportions of CD45RA^-^ CD4^+^ T cells were protective (OR = 0.89, 95% CI = 0.82–0.97, P = 0.009; **Figure 2C**). Complete IVW estimates for all significant metabolite, GM and immune traits, together withsensitivity analyses, are summarized in **Supplementary Table 2**.

These MR findings nominate systemic metabolic, microbial and immune traits that may be relevant to DIE susceptibility; however, in the absence of formal mediation analyses, longitudinal sampling or experimental perturbation, they should be interpreted as hypothesis-generating rather than mechanistically proven.

### 3.3 Colocalization analysis

Colocalization analysis was performed for exposure traits with robust MR associations. For eachprioritized exposure, gene-centered loci were interrogated to determine whether the exposure and DIE shared the same causal variant. This analysis identified 423 exposure–gene pairs with posterior probability for a shared causal variant (PP.H4) > 0.50, corresponding to 324 unique protein-coding genes. Exposure-linked assignments included 198 metabolite-linked, 109 immune-linked and 19 GM-linked genes; because some genes mapped to more than one exposure domain, these categories were not mutually exclusive. These colocalized genes formed the candidate set for subsequent differential expression, functional annotation and modeling (**Supplementary Table 3**). Representative regional colocalization plots for the five feature genes used in the modelare shown in **Supplementary Figure 1**.

### 3.4 Differential expression analysis and differentially expressed associated gene identification

**Figure 3:**
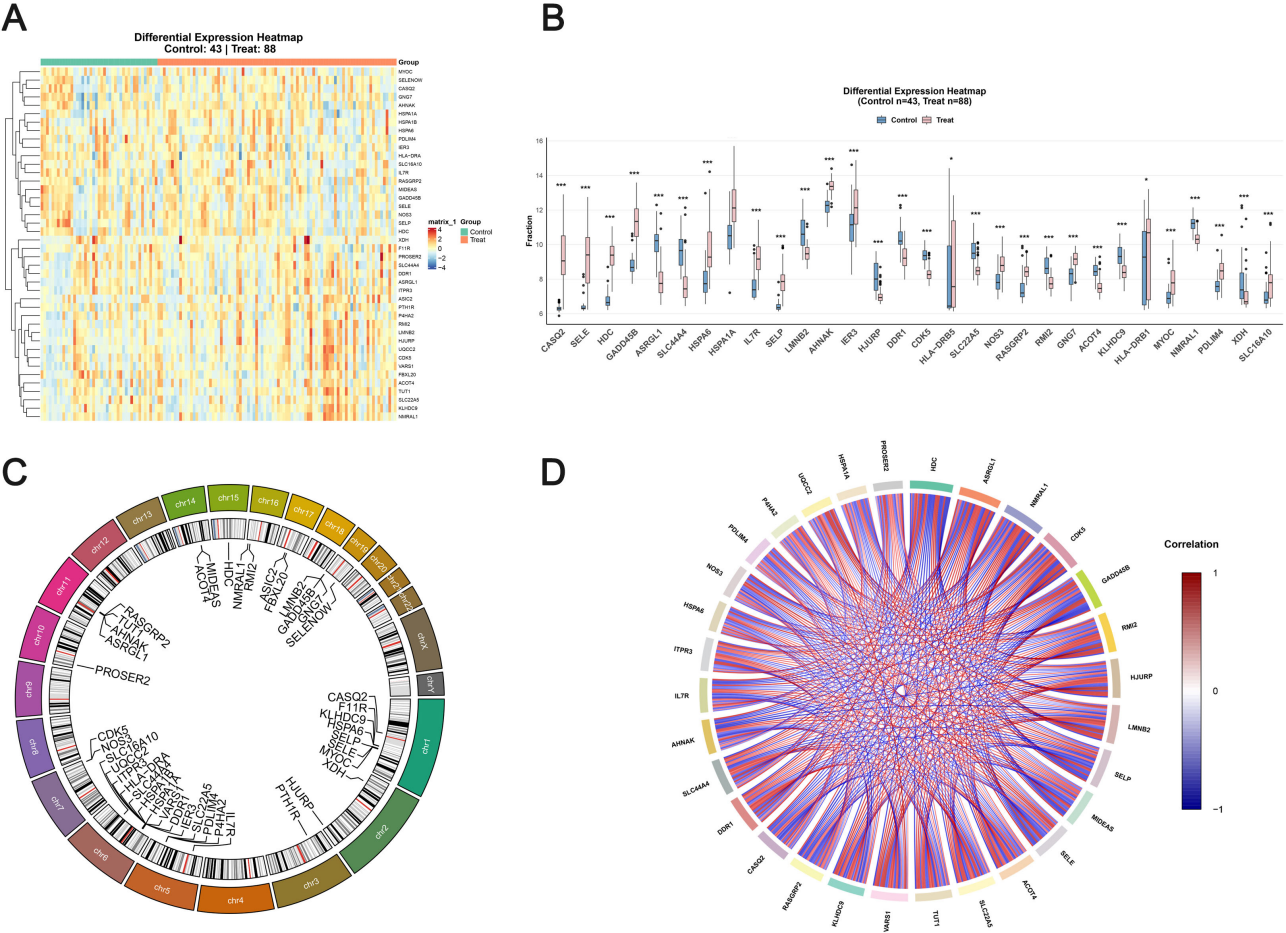
DEAGs, Differentially expressed associated genes between normal endometrium and DIE lesions. **(A)** Heatmap of normalized expression of 42 colocalized DEAGs in GSE141549. **(B)** Boxplots of representative DEAGs comparing normal endometrium and DIE lesions. **(C)** Circos plot of chromosomal distribution of DEAGs. **(D)** Correlation network of DEAGs based on pairwise Spearman coefficients. * indicates P < 0.05, and *** indicates P < 0.001.

Differential expression analysis of colocalized genes between normal endometrium and DIE identified 42 genes with significant expression changes (adjusted P < 0.05), hereafter referred to as differentially expressed associated genes (DEAGs) (**Figure 3A**). These genes showed heterogeneous expression patterns, with 20 upregulated and 22 downregulated in DIE lesions. HDC exhibited the most extreme statistical significance (P = 1.36 × 10⁻^4^⁰) together with marked upregulation (log₂ fold change = 2.55). Other prominently upregulated DEAGs included GADD45B (P = 1.92 × 10⁻²^6^, log₂FC = 2.44) and CASQ2 (P = 1.77 × 10⁻²⁵, log₂FC = 3.07), whereas CDK5 (P = 3.62 × 10⁻²^8^, log₂FC = −1.08), ASRGL1 (P = 7.42 × 10⁻²^4^, log₂FC = −2.22) and SLC44A4 (P = 1.47 × 10⁻¹^7^;, log₂FC = −1.97) were significantly downregulated (**Figure 3B**). The five feature genes selected for modeling—HDC, GADD45B, CDK5, AHNAK and RASGRP2—were all among the most strongly dysregulated DEAGs, supporting their prioritization as candidate lesion-level effectors or correlates consistent with exposure-anchored DIE biology.

Genomic mapping showed that DEAGs were distributed across multiple autosomes rather than clustering within a single chromosomal region, suggesting that DIE-related transcriptional perturbations arise from coordinated effects at dispersed loci (**Figure 3C**). Correlation analysis revealed predominantly positive co-expression among DEAGs, with dense networks linking genes involved in inflammatory signaling, immune regulation and extracellular matrix remodeling (e.g. HDC, GADD45B, IL7R, HSPA1A/HSPA1B/HSPA6, SELP) (**Figure 3D**). Comprehensive differential expression statistics for all 42 DEAGs are provided in **Supplementary Table 4**. We performed functional enrichment via Gene Ontology, KEGG and Reactome. The significantlyenriched functions include hemostasis, immune system, MAPK signaling pathway, Protein processing inendoplasmic reticulum and response to various stimulus. All these enriched functions are associated with DIE. The enrichment results are provided in **Supplementary Figure 3**.

### 3.5 Immune cell infiltration analysis

**Figure 4:**
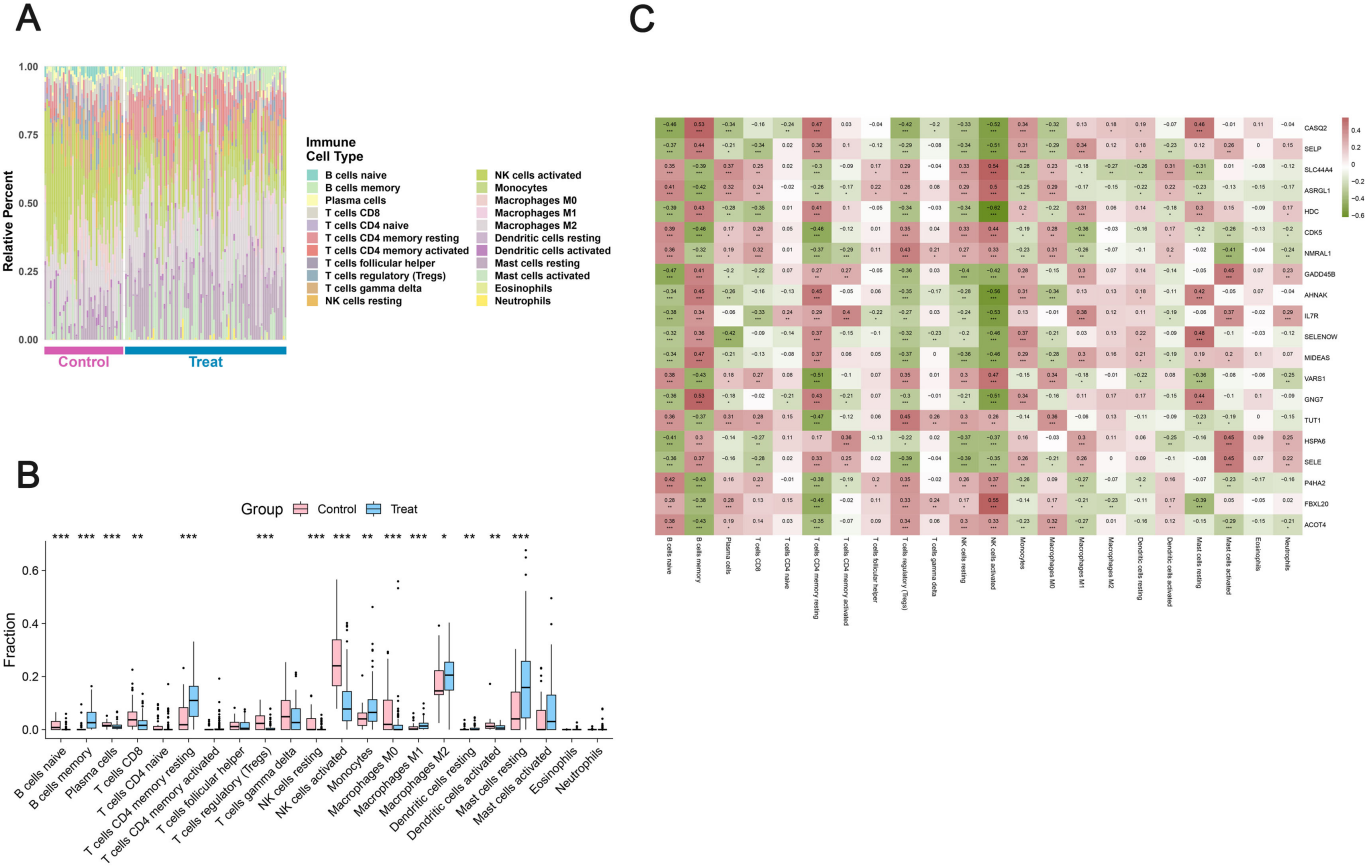
Immune cell infiltration and gene–immune correlations in DIE. **(A)** Stacked barplot of relative fractions of 22 immune cell types inferred by CIBERSORT in normal endometrium and DIE lesions. **(B)** Boxplots of group differences in immune cell fractions. **(C)** Heatmap of Spearman correlations between DEAG expression and immune cell proportions. * indicates P < 0.05, ** indicates P < 0.01, and *** indicates P < 0.001.

Immune cell composition analysis revealed marked differences between normal endometrium and DIE lesions across the 22 immune cell types deconvoluted by CIBERSORT (**Figure 4A**). DIE samples showed a pronounced reshaping of the adaptive immune compartment: memory B cells were significantly enriched, whereas naive B cells were depleted (both *P* < 0.001). Plasma cells were modestly but significantly reduced. Among T-cell subsets, CD8^+^ T cells and regulatory T cells (Tregs) were significantly decreased, whereas CD4^+^ memory resting T cells were substantially increased, indicating a shift from cytotoxic and regulatory toward memory CD4^+^ T-cell–dominated profiles (**Figure 4B**).

Innate immune populations also displayed striking alterations. Both resting and activated naturalkiller (NK) cells were markedly reduced in DIE lesions (*P* < 0.001), suggesting attenuated NK-cell–mediated surveillance. In contrast, myeloid and mast-cell populations were expanded: monocytes, macrophages (particularly M1 and M2 subsets), resting dendritic cells and resting mast cells all showed higher fractions in DIE than in controls (*P* < 0.05 for each), whereas activated dendritic cells were decreased. Neutrophils and several less abundant subsets showed no statistically significant changes. Group-wise differences in immune cell fractions are summarized in **Supplementary Table 5**.

Correlation analysis between DEAGs and immune cell populations revealed structured relationships linking exposure-related transcriptional changes to the altered immune milieu (**Figure 4C**). The five feature genes*-*HDC, GADD45B, CDK5, AHNAK andRASGRP2—showed particularly strong associations with B-cell, NK-cell and CD4^+^ memory T-cell subsets. For example, HDC and AHNAK were strongly negatively correlated with activated NK cells, GADD45B was negatively correlated with naive B cells, and CDK5 showed negative correlations with both memory B cells and CD4^+^ memory resting T cells. RASGRP2 exhibited coordinated correlations with B-cell and mast-cell populations. Comprehensive gene–immune correlation statistics are provided in **Supplementary Table 6**.

### 3.6 Predictive modeling selection and nomogram construction

**Figure 5:**
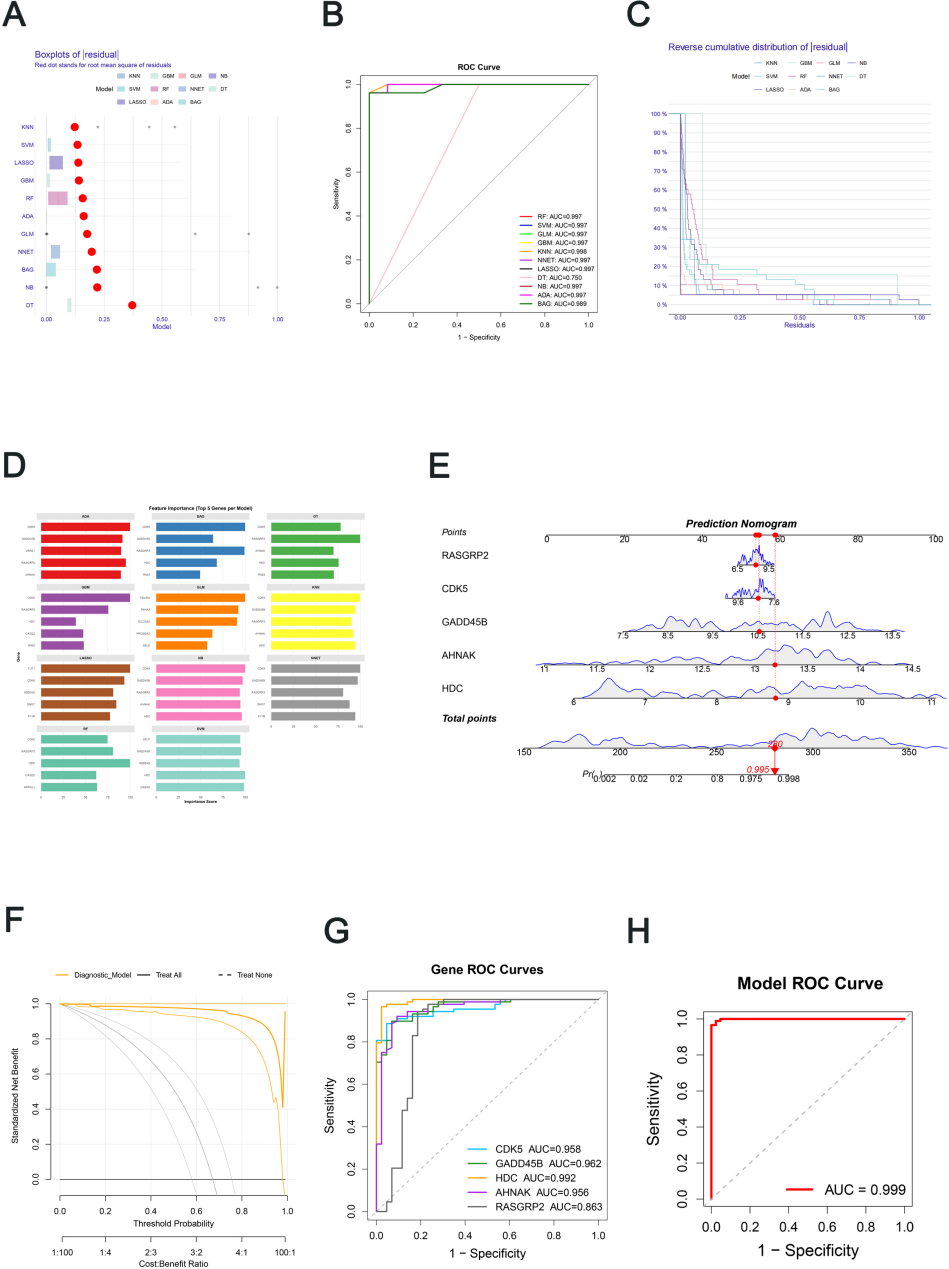
Machine-learning model comparison and five-gene tissue-level classifier nomogram. **(A–C)** Model residuals, ROC curves and residual distributions for eleven machine-learning algorithms based on DEAG expression. **(D)** Feature-importance rankings across algorithms. **(E)** Nomogram of the five-gene logistic regression model. **(F)** Decision curve analysis of the five-gene model. **(G)** ROC curves for each feature gene. **(H)** ROC curve for the combined five-gene model.

Eleven machine-learning algorithms (random forest, support vector machine, generalized linear model, gradient boosting, k-nearest neighbor, neural network, LASSO logistic regression, decision tree, naïve Bayes, AdaBoost and bagging) were evaluated for distinguishing DIE lesions from normal endometrium based on DEAG expression profiles. Cross-validated comparisons indicated that the k-nearest neighbor (KNN) model provided a favorable overall balance of discrimination and error stability, and, in particular, a robust and consistent feature-importance ranking across resampling. Residual and reverse cumulative residual plots further supported the stability of KNN-based predictions across samples (**Figures 5A–C**). Feature-importance profiles across algorithms, and especially from the KNN model, consistently highlighted a small subset of genes as major contributors to classification, supporting the selection of five feature genes—HDC, GADD45B, CDK5, AHNAK and RASGRP2—for subsequent tissue-level modeling (**Figure 5D**; **Supplementary Table 7**).

A five-gene logistic regression model was then fitted and visualized as a nomogram to provide quantitative within-cohort estimates of DIE lesion classification (**Figure 5E**). Each feature gene contributed a specific point value according to its expression level, and the total point score corresponded to the predicted probability of DIE. Internal evaluation within the GSE141549 cohort demonstrated excellent discrimination (AUC = 0.999; **Figure 5H**) and close agreement between predicted and observed probabilities, with calibration curves closely tracking the ideal reference line.

Decision curve analysis suggested potential within-cohort decision-analytic value of the five-gene model across a range of threshold probabilities (**Figure 5F**). When evaluated individually, each feature gene also exhibited strong discriminatory performance, with HDC achieving the highest AUC (0.992), followed by GADD45B (0.962), CDK5 (0.958), AHNAK (0.956) and RASGRP2 (0.863) (**Figure 5G**; **Supplementary Table 8**). A non-significant Hosmer−Lemeshow test supported acceptable internal calibration. Together, these findings indicate that the five genes capture substantial discriminative information between DIE lesions and normal endometrium within this cohort; however, external validation and comparison against established clinical predictors are required before translational utility can be claimed.

### 3.7 Immunohistochemical validation of feature genes

**Figure 6:**
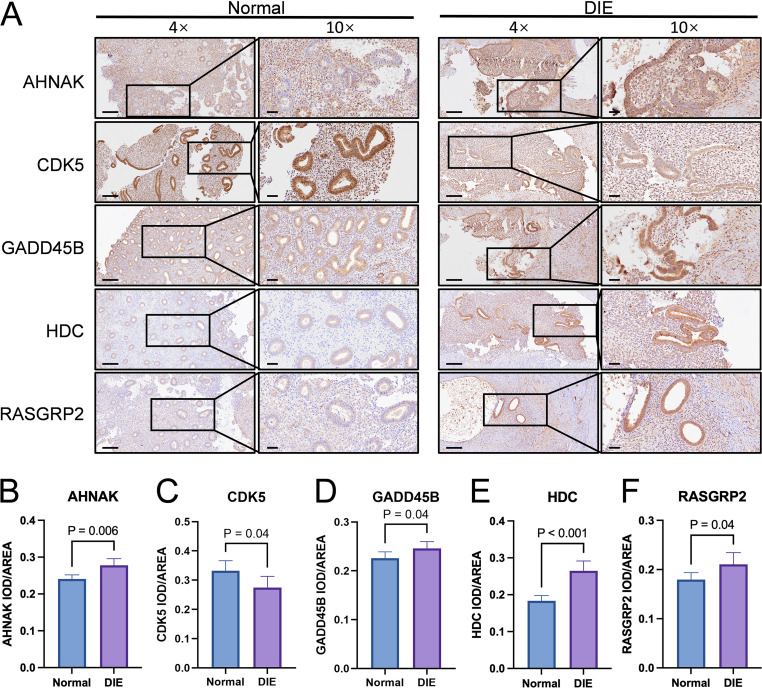
Immunohistochemical validation of the five feature genes in clinical specimens. **(A)** Immunohistochemical staining of AHNAK, CDK5, GADD45B, HDC and RASGRP2 from normal endometrium and DIE lesions at 4× and 10× magnification, with enlarged insets highlighting glandular epithelium and stroma. Images are shown at 4× (scale bar = 200 μm) and 10× (scale bar = 50 μm). (**B**–**F**) Bar graphs on the right show quantitative comparison of IOD/area values for each marker between normal and DIE tissues.

Representative IHC images at 4× and 10× magnification (**Figure 6A**) showed clear spatial differences in staining patterns for the five feature genes between normal endometrium and DIE lesions. In normal tissue, AHNAK, GADD45B and RASGRP2 staining was mainly localized to glandular epithelial cells with only weak labelling of the surrounding stroma, whereas in DIE lesions these markers displayed stronger and more diffuse staining in both the glandular epithelium and fibromuscular stromal compartment. HDC staining in normal endometrium was largely restricted to scattered stromal cells, while DIE lesions showed a marked increase in HDC-positive stromal and perilesional cells around glands. By contrast, CDK5 showed intense membranous/cytoplasmic staining along normal glandular epithelium, which was visibly attenuated in the epithelium of DIE lesions, with only faint and patchy stromal staining.

Quantitative ImageJ analysis of IOD/area confirmed these visual impressions: CDK5 protein expression was significantly lower in DIE tissues than in normal endometrium (*P* = 0.04; **Figure 6C**), whereas AHNAK, GADD45B, HDC and RASGRP2 were all significantly upregulated in DIE (P = 0.006, *P* = 0.04, *P* < 0.001 and *P* = 0.04, respectively; **Figures 6B, D–F**). Among these, HDC showed the most pronounced increase. Overall, the direction of protein-level changes and their epithelial–stromal distribution closely mirrored the transcriptomic differences observed in GSE141549 and were consistent with the effects inferred from MR and predictive modeling, providing orthogonal validation of the five exposure-related feature genes in DIE.

## 4 Discussion

In this study, we integrated Mendelian randomization, Bayesian colocalization, bulk RNA sequencing, immune-cell deconvolution, machine-learning–based feature prioritization and immunohistochemistry to define a putative gut–immune–pelvic pathway in deep infiltrating endometriosis (DIE). By linking genetically proxied systemic exposures to lesion-level molecular changes, our framework generates biologically plausible hypotheses about how metabolic, microbial and immune traits may converge on a fibrotic pelvic microenvironment (2, 21).

Our MR analyses pointed to several metabolite classes*-*including bile-acid–related species, acylcarnitines and selected amino-acid derivatives*-*as candidate contributors to DIE risk, alongside lipid species with apparently protective effects. These findings are concordant with targeted and untargeted metabolomics studies reporting broad disturbances in amino-acid, lipid and organic-acid metabolism in serum and peritoneal fluid from women with moderate-to-severe endometriosis, with enrichment of glycolytic and tricarboxylic-acid-cycle intermediates and altered acylcarnitine and phospholipid species indicative of mitochondrial stress and oxidative imbalance (4). Because the metabolite instruments were derived from a large whole-genome–sequencing-based GWAS of 1,666 plasma metabolites (11), our results suggest that at least part of this metabolic signature is rooted in germline variation rather than being solely a secondary consequence of chronic pain, hormonal treatment or lifestyle factors.

The gut microbiota findings extend the emerging concept of a gut–endometriosis pathway to a DIE-specific context. Genetically proxied higher abundances of taxa such as Mycobacteriaceae and UBA7182 were associated with increased risk, whereas genera including Faecalicatena and Succinivibrio showed protective profiles. These patterns are in line with human and experimental data indicating that endometriosis is accompanied by reduced microbial diversity, depletion of short-chain-fatty-acid–producing bacteria, expansion of pro-inflammatory taxa and increased microbial β-glucuronidase activity that can enhance enterohepatic recycling of estrogens and promote mucosal inflammation (5). A parsimonious interpretation is that dysbiosis may modulate both estrogen bioavailability and low-grade systemic inflammation, thereby lowering the threshold for ectopic implantation and favoring progression toward deeply infiltrative, fibrotic disease.

In parallel, MR signals for immune traits*-*particularly monocytic and dendritic-cell phenotypes that increased risk, and CD45RA^-^ CD4^+^ T-cell subsets that appeared protective—fit with current models of an endometriosis-associated immune milieu characterized by activated, cytokine-producing macrophages, altered T-cell compartmentalization and impaired natural killer (NK) cell cytotoxicity in the peritoneal cavity (3, 22, 23). Together, these observations support a working model in which host metabolism, gut microbes and the immune system interact to shape inter-individual differences in susceptibility to DIE.

One biologically plausible route linking the metabolite- and microbiota-related MR signals to DIE-specific pelvic pathology involves the gut barrier–estrogen–immune interface. Dysregulated bile-acid pools may alter intestinal permeability and receptor-mediated immune signaling, thereby facilitating low-grade endotoxaemia and activation of macrophages and mast cells, which in turn can amplify TGF-β-, IL-6- and TNF-α-rich pro-fibrotic signaling within the pelvic microenvironment. At the same time, depletion of short-chain-fatty-acid–associated genera may weaken epithelial barrier maintenance and anti-inflammatory immune tone, whereas taxa with higher β-glucuronidase activity may enhance enterohepatic estrogen recycling. Together, these processes could lower the threshold for ectopic implantation, sustain immune-cell recruitment, and promote extracellular-matrix deposition and lesion fibrosis in DIE. We emphasize that this cascade remains a hypothesis-generating interpretation rather than a mechanistically proven sequence, but it provides a biologically plausible explanation for how systemic bile-acid and microbial traits may be coupled to deeply infiltrative pelvic disease (24, 25).

By integrating MR with colocalization, we refined exposure-level associations to 324 protein-coding genes with evidence of shared causal variants between exposures and DIE, and then further restricted this set to 42 genes that were differentially expressed in DIE lesions relative to normal endometrium. These differentially expressed associated genes were enriched in pathways related to inflammatory signaling, cellular stress responses and extracellular-matrix (ECM) remodeling, processes that are central to the dense fibrosis, smooth-muscle metaplasia and neuroangiogenesis that typify DIE nodules (21). This stepwise strategy mitigates an important limitation of MR analyses based solely on summary statistics—namely, that statistically robust instruments may act through distal regulatory mechanisms that are not active in the tissue of interest—by requiring that colocalized genes also show transcriptional perturbation in disease tissue.

Within this gene set, the five feature genes prioritized for modeling—HDC, GADD45B, CDK5, AHNAK and RASGRP2—should not be interpreted as a fully validated regulatory network. Rather, they emerged at the intersection of genetic prioritization, lesion-level differential expression, structured gene–immune correlations and protein-level validation, and therefore capture complementary aspects of DIE lesion biology. HDC encodes histidine decarboxylase, the rate-limiting enzyme for histamine synthesis. Mast-cell accumulation, increased histamine levels and intimate contacts between mast cells, sensory nerve fibers and endometriotic glands have been reported in peritoneal and deeply infiltrating lesions, where histamine signaling is thought to contribute to pain, angiogenesis and fibroblast activation (26). Upregulation of HDC in DIE lesions, together with its association with specific immune-cell subsets in our data, is therefore consistent with a histamine-rich, pro-fibrotic microenvironment.

GADD45B is a stress-response gene induced by inflammatory and genotoxic stimuli, and studies in liver and other organs have implicated GADD45 family members in the regulation of apoptosis, cellular senescence and fibrogenesis (27). Its strong upregulation and genetic prioritization in our analysis suggest that chronic inflammatory stress responses may be integral to the maintenance of DIE lesions, although direct functional data in endometriosis are not yet available. AHNAK, a large scaffolding protein, has been shown to modulate TGF-β/Smad3 signaling and to promote epithelial–mesenchymal transition and invasive behavior in cancer and fibrotic models (28). The marked increase of AHNAK expression in DIE, together with its co-expression with ECM- and inflammation-related genes, is compatible with a role in the EMT/fibroblast-to-myofibroblast transition programs that underpin contractility and deep tissue invasion.

CDK5 is best known for its functions in the nervous system, where it regulates cytoskeletal dynamics, neuronal excitability and nociceptive signaling, but accumulating evidence also links CDK5 to inflammatory responses and cell motility outside the central nervous system (29). The combination of a protective MR profile and reduced CDK5 expression in DIE lesions raises the possibility that loss of CDK5 activity may favor aberrant innervation or altered stromal cell behavior in fibrotic nodules, hypotheses that merit experimental testing. RASGRP2 (also known as CalDAG-GEFI) is a guanine nucleotide exchange factor for Rap1 that is essential for integrin activation in platelets and leukocytes, and germline loss-of-function mutations cause bleeding disorders and impaired leukocyte adhesion (30). In the context of DIE, higher RASGRP2 expression may link metabolite- and microbiota-driven platelet and immune activation to endothelial adhesion, microthrombosis and vascular remodeling within lesions.

The immune-cell landscape inferred from GSE141549 is coherent with this putative pathway framework. Compared with normal endometrium, DIE lesions showed enrichment of memory B cells, monocytes, macrophages (including both M1- and M2-like subsets), resting dendritic cells and resting mast cells, with concomitant reductions in CD8^+^ T cells, regulatory T cells and both resting and activated NK cells. Similar shifts toward activated macrophages, dysfunctional NK cells and skewed T-cell responses have been described in peritoneal fluid and ectopic lesions of women with endometriosis (3). The structured correlations observed between feature-gene expression and specific immune subsets—for example, negative associations of HDC and AHNAK with activated NK cells, and coordinated relationships between RASGRP2 and B-cell and mast-cell populations—therefore suggest that these genes may participate in, rather than merely reflect, the immune reprogramming that accompanies DIE.

From a translational perspective, the five-gene logistic model derived from our multi-omics pipeline showed excellent internal discrimination between DIE lesions and normal endometrium in the GSE141549 cohort, together with acceptable internal calibration. Although the near-perfect AUC is likely influenced by the homogeneous case definition (deep lesions only) and by the use of high-quality RNA-seq rather than more heterogeneous clinical specimens (12), the model illustrates how exposure-anchored transcriptomic signatures can capture pathophysiologically meaningful information beyond conventional histopathology. Importantly, we validated the direction of transcriptional changes at the protein level in independent clinical specimens, confirming upregulation of HDC, GADD45B, AHNAK and RASGRP2 and downregulation of CDK5 by immunohistochemistry in DIE versus normal endometrium. The convergence of genetic, transcriptomic and protein-level evidence strengthens the rationale for considering these genes as a composite biomarker panel and as entry points for mechanistic studies.

Our study, integrating Mendelian randomization, colocalization, tissue transcriptomics, and immune deconvolution, systematically delineates a gut–immune–pelvic pathway in DIE, which is consistent with and complementary to several recent multi−omics investigations. Chen et al. (2025) used multiomic Mendelian randomization and demonstrated that cell aging−related pathways, including oxidative stress, apoptosis, and inflammatory signaling, contribute to endometriosis pathogenesis, highlighting links between cellular senescence, chronic inflammation, and fibrotic microenvironments (31). Our findings are highly concordant: key genes identified here, including GADD45B and AHNAK, are strongly involved in cellular stress, senescence, and fibrosis and are markedly dysregulated in DIE lesions, jointly supporting that aberrant cellular stress and senescence programs are pivotal pathogenic mechanisms in endometriosis. Guo et al. (2025) applied two−sample MR and confirmed causal links and mediating effects of lipid metabolism, immune cells, and inflammatory proteins in endometriosis, highlighting pathogenic roles of triglycerides, apolipoproteins, and imbalanced myeloid/lymphoid subsets (32). We further anchored three−layer multi−omics (metabolome, microbiome, immunome) to DIE−specific causal associations and identified HDC, CDK5, and RASGRP2 as key effector genes that directly connect metabolic/immune signals to lesion invasion and fibrosis, thus refining the molecular pathways linking systemic metabolic−immune disturbances to local pelvic pathology. Ou et al. (2025) provided multi−omics insights into endometriosis−associated infertility, highlighting connections among metabolic reprogramming, immune dysregulation, and impaired reproductive function, which informs precision therapeutics (33). By focusing on the most aggressive DIE subtype, we established and validated a five−gene diagnostic panel at the protein level, complementing the work of Ou et al. and jointly supporting that multi−omics signatures can aid disease subtyping, risk stratification, and targeted intervention development.

Several limitations should be acknowledged. First, the MR analyses relied on GWAS summary statistics predominantly from individuals of European ancestry, which may limit generalizability and leaves residual confounding from population structure impossible to exclude completely (8–10). Second, although MR and colocalization provide genetically anchored evidence, formal mediation analysis was not performed; therefore, we cannot determine whether metabolite or microbiota signals influence DIE directly or indirectly through immune remodeling. Third, the lesion transcriptomic, immune-deconvolution and modeling analyses were based on a single cross-sectional RNA-seq dataset comparing DIE lesions with normal endometrium, and detailed menstrual-cycle and lesion-composition information was limited; independent replication will therefore be important (12). Fourth, the five-gene model should be interpreted as an internally evaluated tissue-level classifier rather than a clinically deployable diagnostic test, and our IHC series was small. Fifth, CIBERSORT-like deconvolution provides only indirect inference of immune-cell composition and cannot substitute for single-cell or flow-cytometric validation.

More broadly, emerging literature supports the notion that endometriosis is not merely an isolated pelvic disorder, but rather is embedded in wider systemic metabolic and inflammatory networks. Recent reviews have increasingly framed endometriosis as a chronic systemic or multisystem disease accompanied by clinically relevant extra-pelvic comorbidities (34, 35). A retrospective matched cohort study further reported elevated risks of multiple chronic comorbidities among women with endometriosis (36). Of particular relevance, a recent prospective cohort study demonstrated that laparoscopically confirmed endometriosis was associated with a higher risk of incident NAFLD (HR 1.17, 95% CI 1.07-1.29) (37). Given that bile acids, intestinal barrier dysfunction, and microbiota-driven immune signaling are key pathways underlying gut-liver and hepatosplenic crosstalk in NAFLD/liver fibrosis biology (38–40), this broader multisystem hypothesis is biologically plausible. Notably, however, our study did not evaluate NAFLD outcomes, liver phenotypes, hepatic biomarkers, or direct markers of gut-liver/hepatosplenic signaling. Our findings should therefore not be interpreted as evidence supporting a generalized organ-connection model, but rather as lending support to a putative gut-immune-pelvic pathway in DIE. Whether endometriosis is mechanistically linked to extra-pelvic metabolic pathways such as the gut-liver axis remains to be elucidated in future dedicated longitudinal and organ-specific studies.

In summary, by integrating MR, colocalization, lesion transcriptomics, immune deconvolution, machine learning and protein-level validation, we provide a framework in which specific circulating metabolites, gut microbial taxa and immune-cell phenotypes may converge on a fibrotic, immune-inflammatory and neuroangiogenic microenvironment in deep infiltrating endometriosis (4, 5, 21). The five-gene panel comprising HDC, GADD45B, CDK5, AHNAK and RASGRP2 emerges as a biologically plausible tissue-level signature consistent with this putative pathway. Extending this framework to diverse populations and mechanistic models may help refine future biomarker and therapeutic research in DIE.

## 5 Conclusion

In summary, this multi-omics Mendelian randomization study links genetically proxied metabolic, microbial and immune traits to deep infiltrating endometriosis and supports a putative gut–immune–pelvic pathway in which systemic perturbations may converge on a fibrotic, immune-inflammatory and neuroangiogenic lesion microenvironment. By integrating colocalization, lesion transcriptomics, immune-cell deconvolution, machine-learning–based feature prioritization and immunohistochemical validation, we identified a five-gene panel—HDC, GADD45B, CDK5, AHNAK and RASGRP2—that represents a biologically plausible tissue-level signature consistent with this framework and shows strong within-cohort discriminatory performance. These findings warrant external validation and mechanistic investigation, rather than establishing a validated bidirectional organ axis or an immediately applicable clinical test.

## Data Availability

The original contributions presented in the study are included in the article/**Supplementary Material**. Further inquiries can be directed to the corresponding authors.
