# Supplementary material for: Multi-omics Mendelian randomization integrating metabolism, microbiome and immunity supports a putative gut-immune-pelvic pathway in deep infiltrating endometriosis
Source: Front Endocrinol (Lausanne). 2026 May 21;17:1827134. doi: 10.3389/fendo.2026.1827134 (PMC13233194; doi:10.3389/fendo.2026.1827134)
Supplement: Supplementary file 11 [file Image1.pdf]

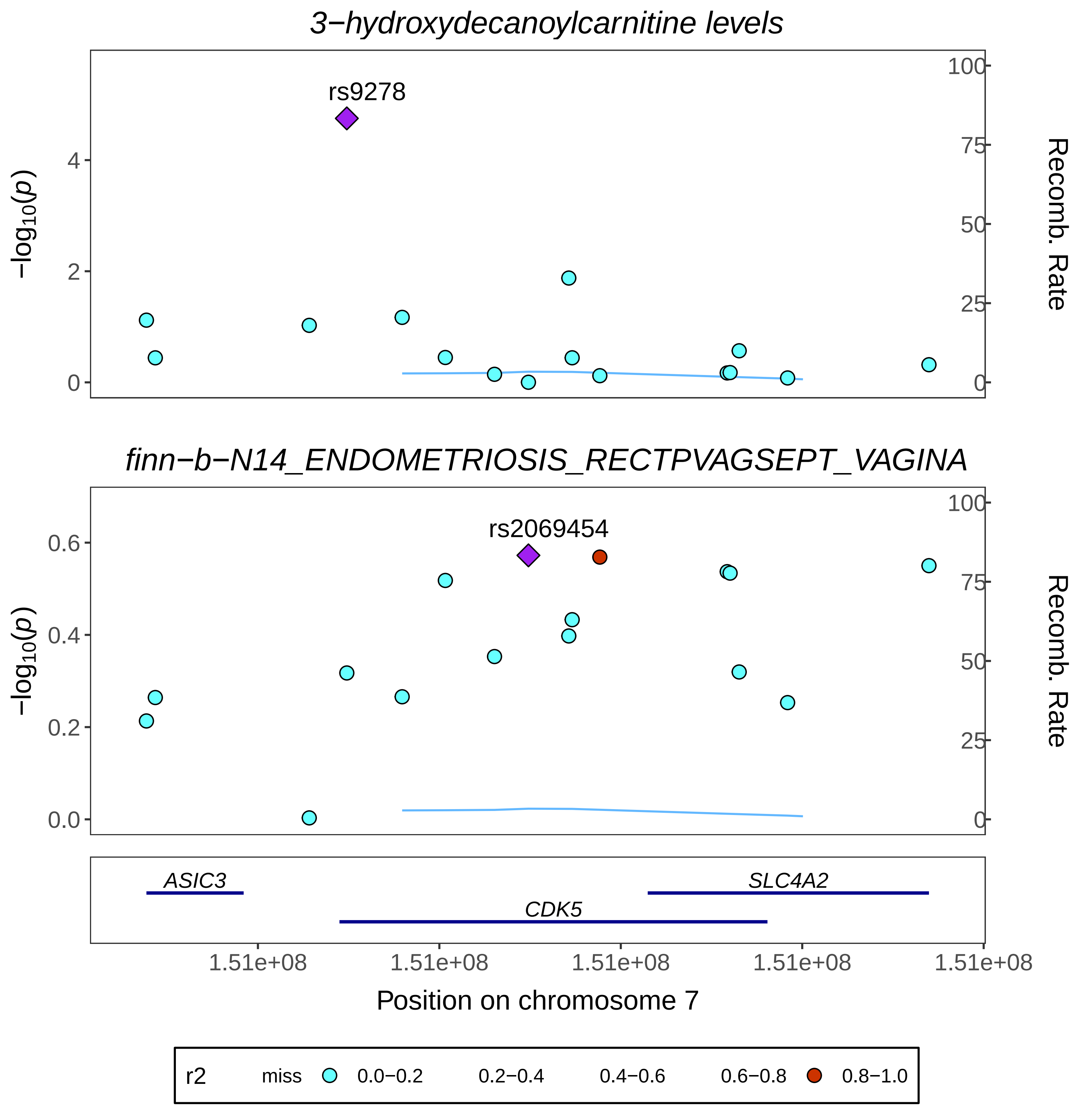
3−hydroxydecanoylcarnitine levels
rs9278
−log10(p)
Recomb. Rate
0
2
4
0
25
50
75
100
finn−b−N14_ENDOMETRIOSIS_RECTPVAGSEPT_VAGINA
rs2069454
0.0
0.2
0.4
0.6
ASIC3
SLC4A2
CDK5
1.51e+08
1.51e+08
1.51e+08
1.51e+08
1.51e+08
Position on chromosome 7
r2
miss
0.0−0.2
0.2−0.4
0.4−0.6
0.6−0.8
0.8−1.0

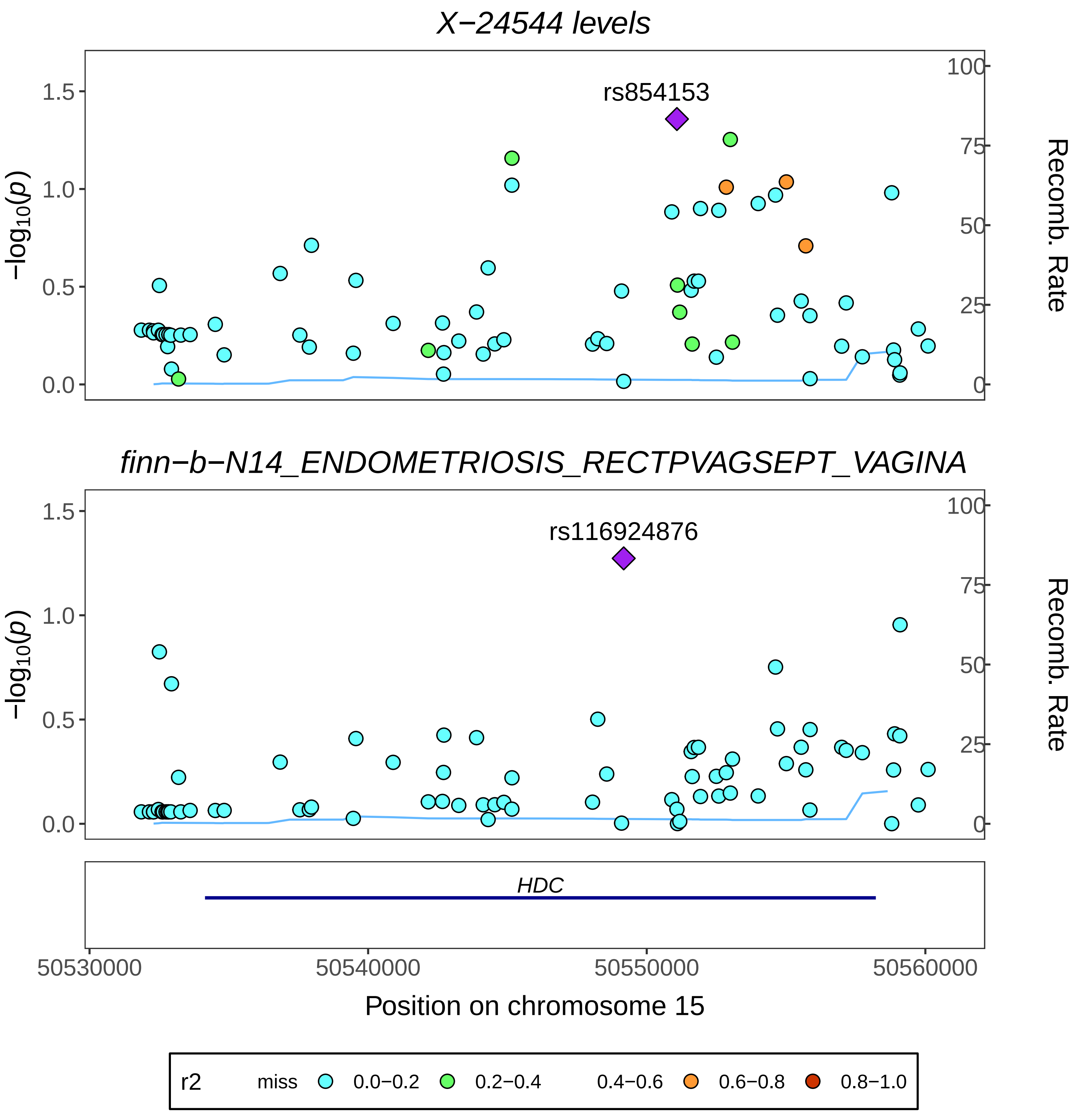

X−24544 levels
rs854153
−log10(p)
Recomb. Rate
finn−b−N14_ENDOMETRIOSIS_RECTPVAGSEPT_VAGINA
rs116924876
HDC
50530000
50540000
50550000
50560000
Position on chromosome 15
r2
miss
0.0−0.2
0.2−0.4
0.4−0.6
0.6−0.8
0.8−1.0

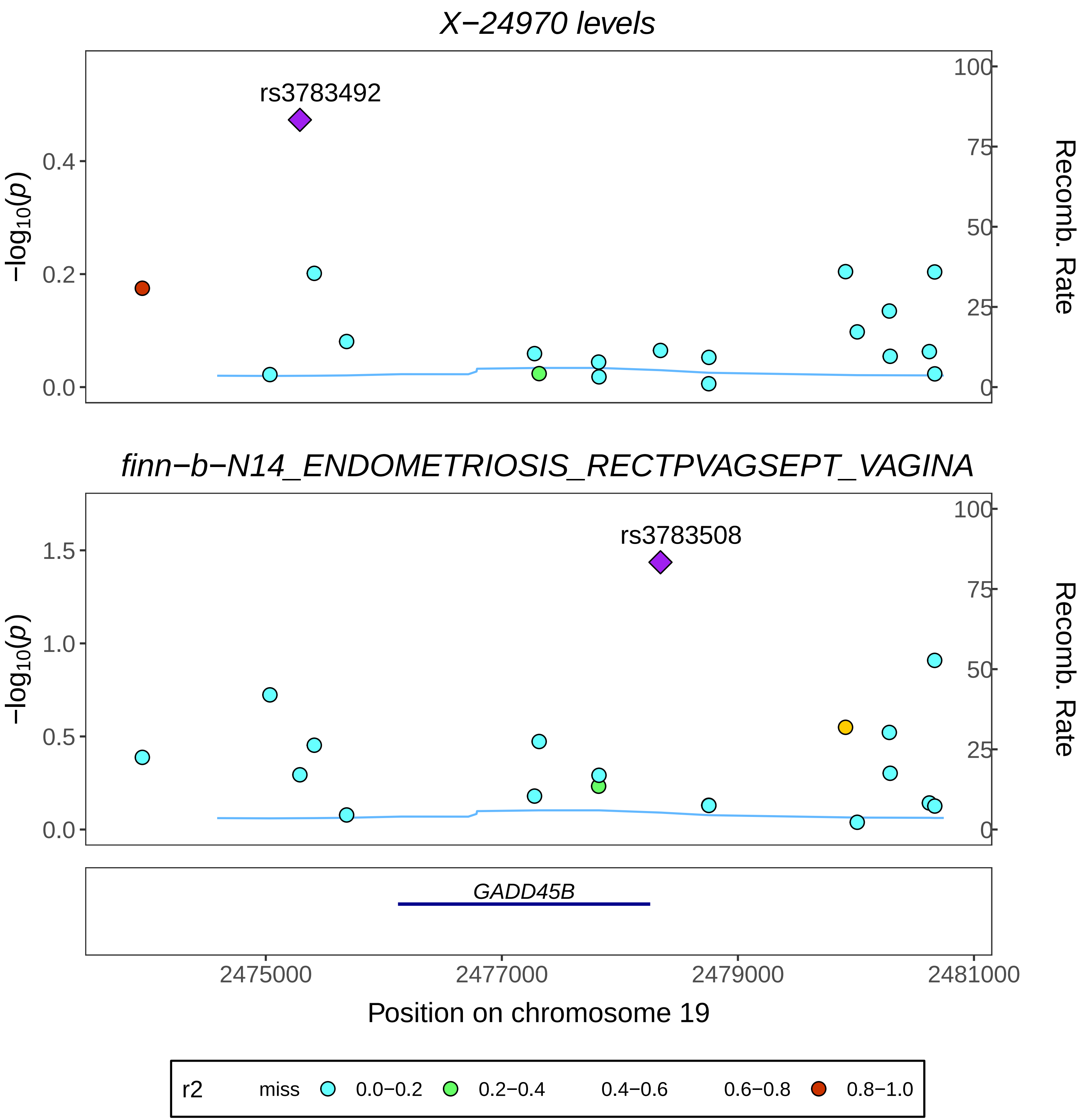
X−24970 levels
rs3783492
−log10(p)
Recomb. Rate
0.0
0.2
0.4
0
25
50
75
100
finn−b−N14_ENDOMETRIOSIS_RECTPVAGSEPT_VAGINA
rs3783508
0.0
0.5
1.0
1.5
GADD45B
2475000
2477000
2479000
2481000
Position on chromosome 19
r2
miss
0.0−0.2
0.2−0.4
0.4−0.6
0.6−0.8
0.8−1.0

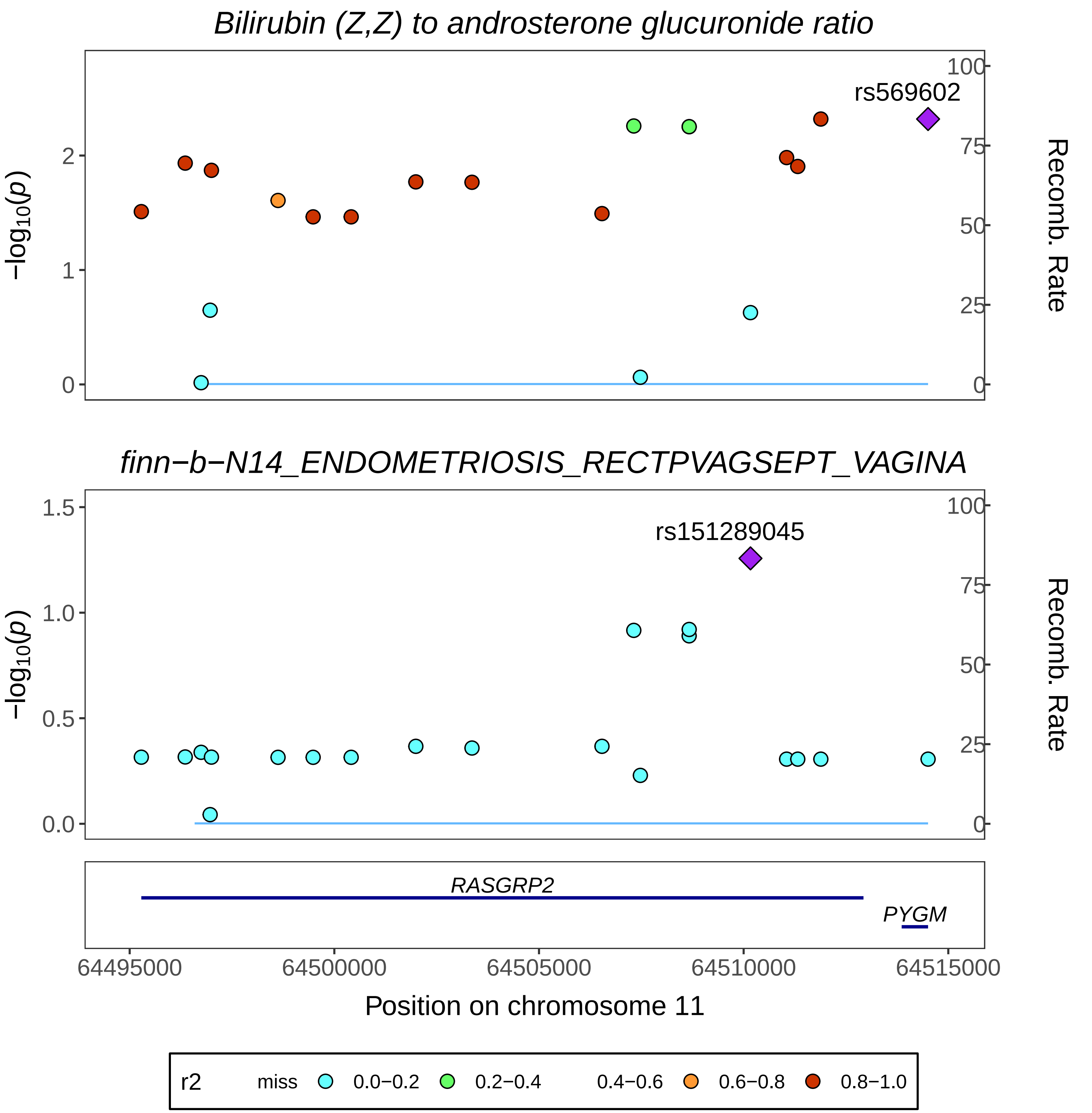

Bilirubin (Z,Z) to androsterone glucuronide ratio
rs569602
−log10(p)
Recomb. Rate
finn−b−N14_ENDOMETRIOSIS_RECTPVAGSEPT_VAGINA
rs151289045
−log10(p)
Recomb. Rate
RASGRP2
PYGM
64495000
64500000
64505000
64510000
64515000
Position on chromosome 11
r2
miss
0.0−0.2
0.2−0.4
0.4−0.6
0.6−0.8
0.8−1.0

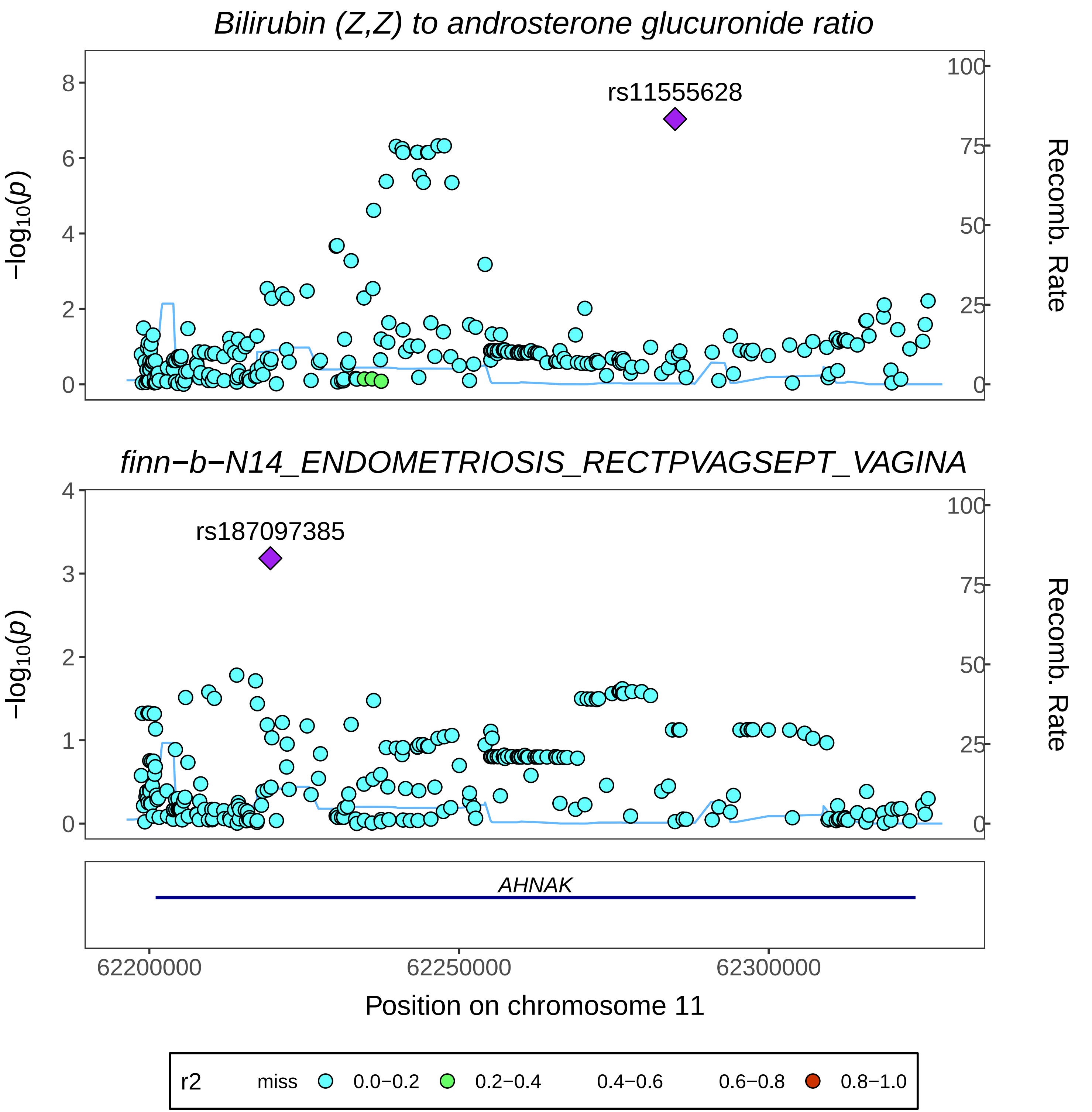

Bilirubin (Z,Z) to androsterone glucuronide ratio
rs11555628
−log10(p)
Recomb. Rate
finn−b−N14_ENDOMETRIOSIS_RECTPVAGSEPT_VAGINA
rs187097385
AHNAK
62200000
62250000
62300000
Position on chromosome 11
r2
miss
0.0−0.2
0.2−0.4
0.4−0.6
0.6−0.8
0.8−1.0
