# Supplementary material for: Multi-omics Mendelian randomization integrating metabolism, microbiome and immunity supports a putative gut-immune-pelvic pathway in deep infiltrating endometriosis
Source: Front Endocrinol (Lausanne). 2026 May 21;17:1827134. doi: 10.3389/fendo.2026.1827134 (PMC13233194; doi:10.3389/fendo.2026.1827134)
Supplement: Supplementary file 12 [file Image2.pdf]

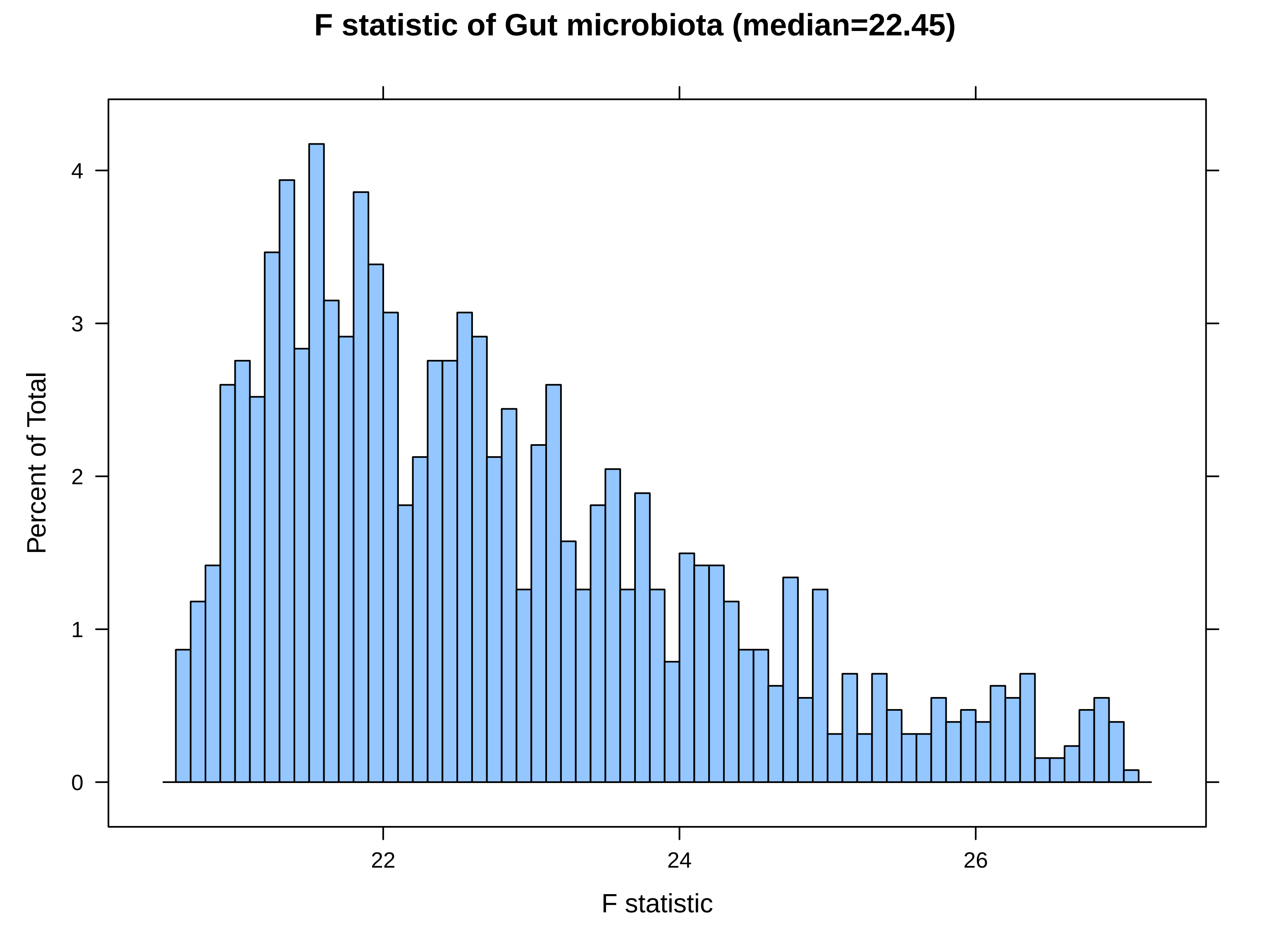
F statistic of Gut microbiota (median=22.45)
Percent of Total
0
1
2
3
4
22
24
26
F statistic

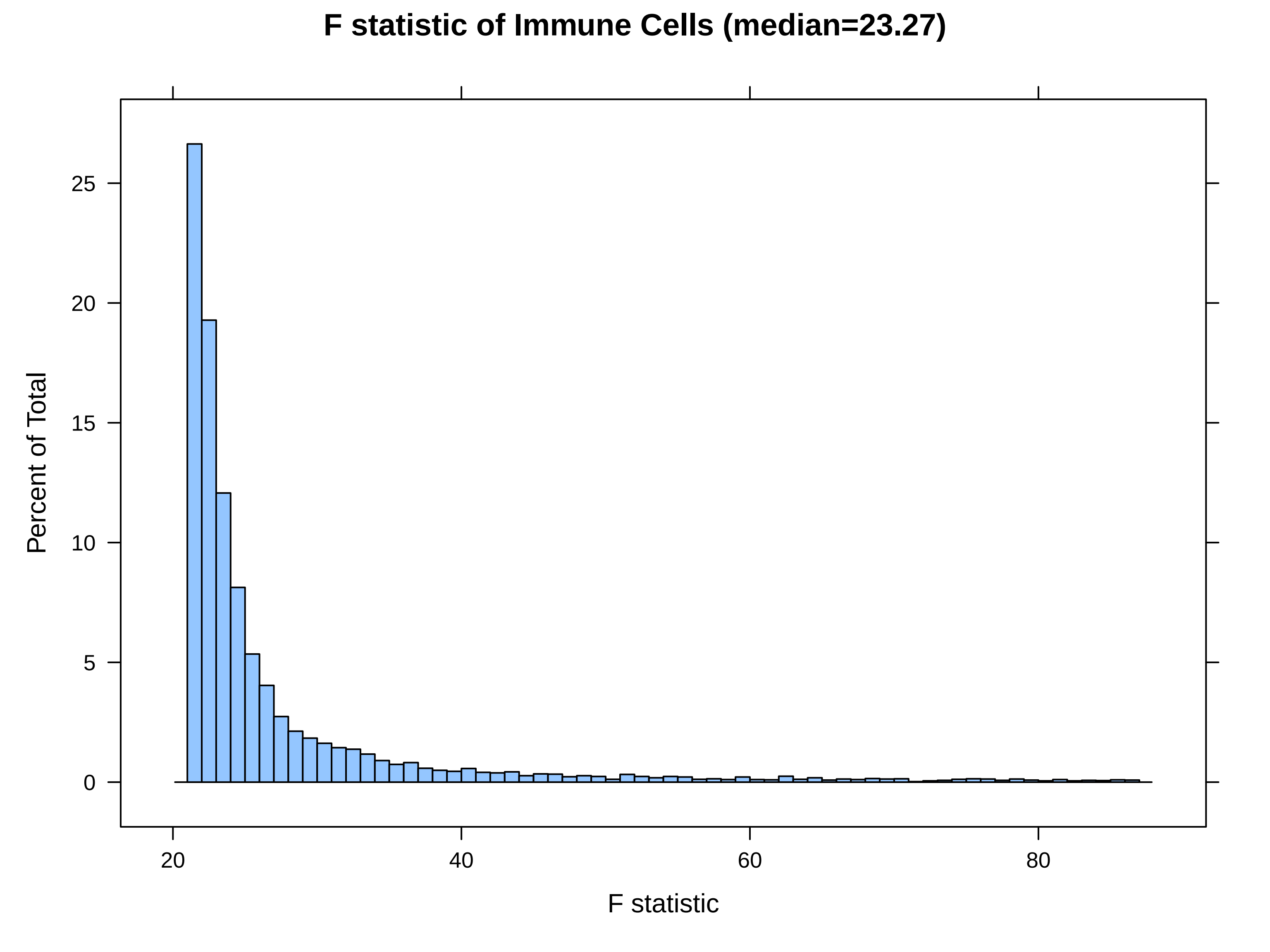
F statistic of Immune Cells (median=23.27)
Percent of Total
0
5
10
15
20
25
20
40
60
80
F statistic

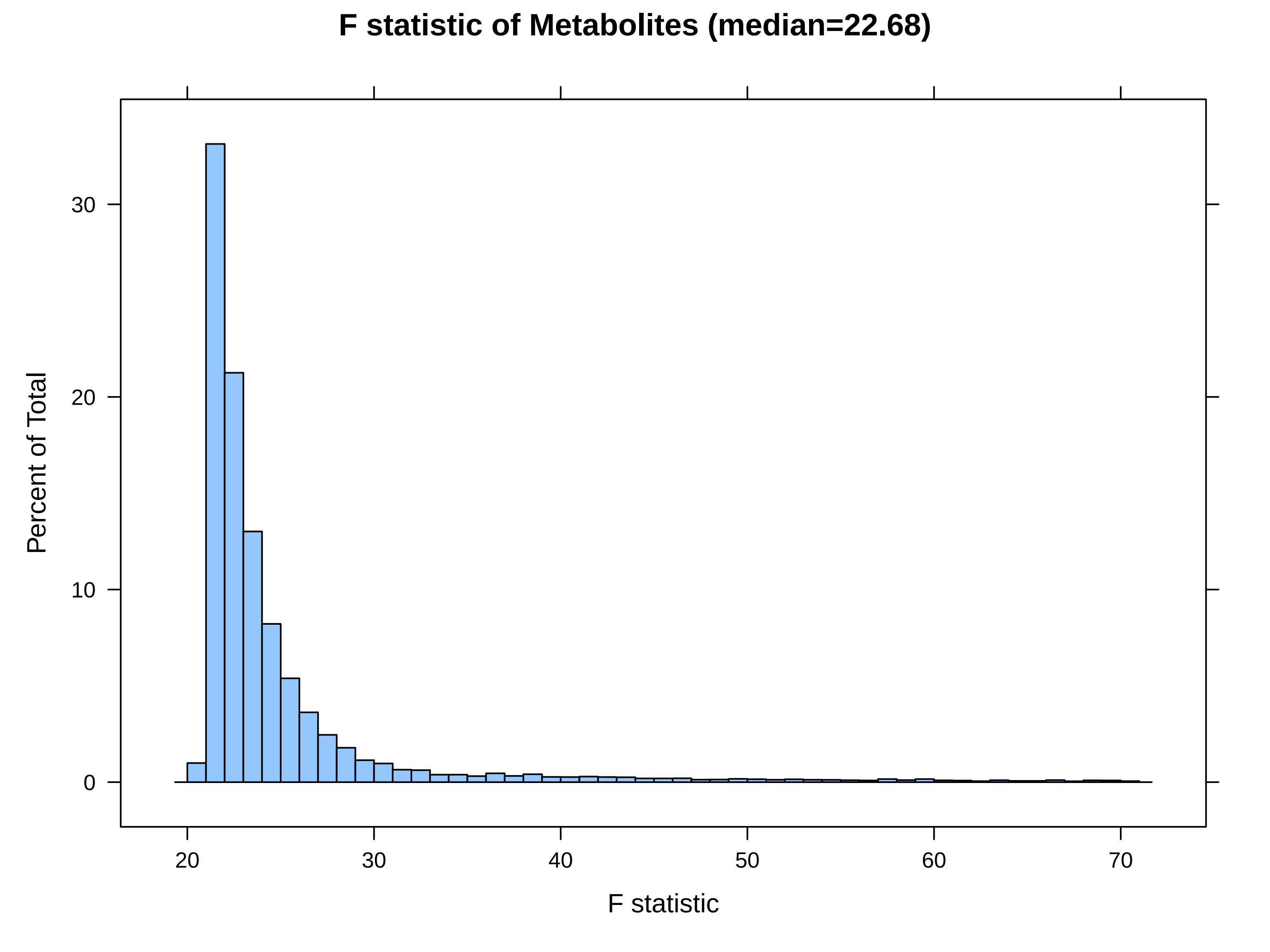
F statistic of Metabolites (median=22.68)
Percent of Total
F statistic
0
10
20
30
20
30
40
50
60
70
