# Supplementary figures and images for: Multi-omics Mendelian randomization integrating metabolism, microbiome and immunity supports a putative gut-immune-pelvic pathway in deep infiltrating endometriosis
Source: Front Endocrinol (Lausanne). 2026 May 21;17:1827134. doi: 10.3389/fendo.2026.1827134 (PMC13233194; doi:10.3389/fendo.2026.1827134)

## GSES of Gene Ontology

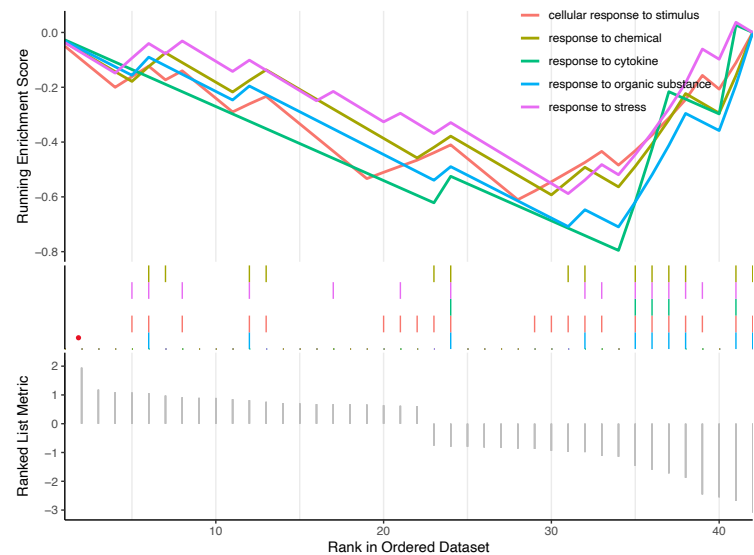

A

## GSES of KEGG

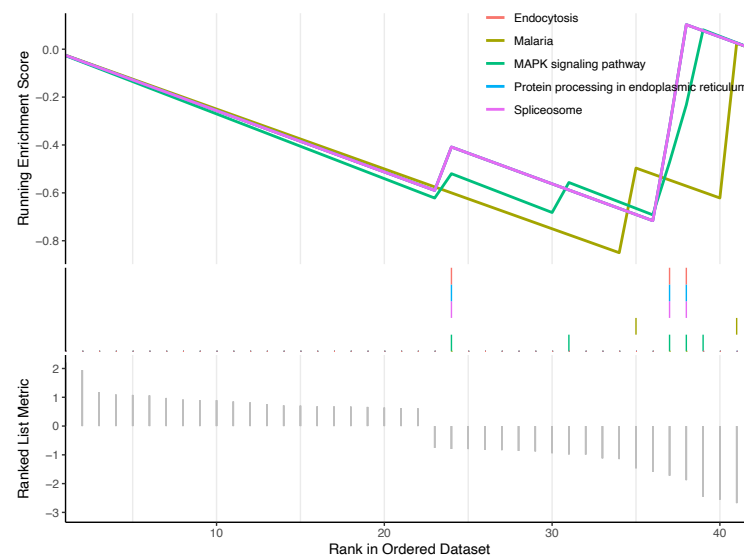

B

## GSES of Reactome

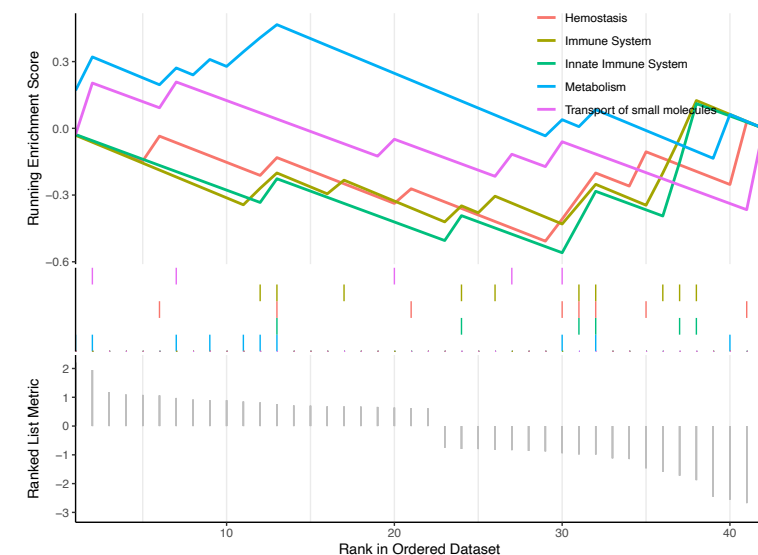

C

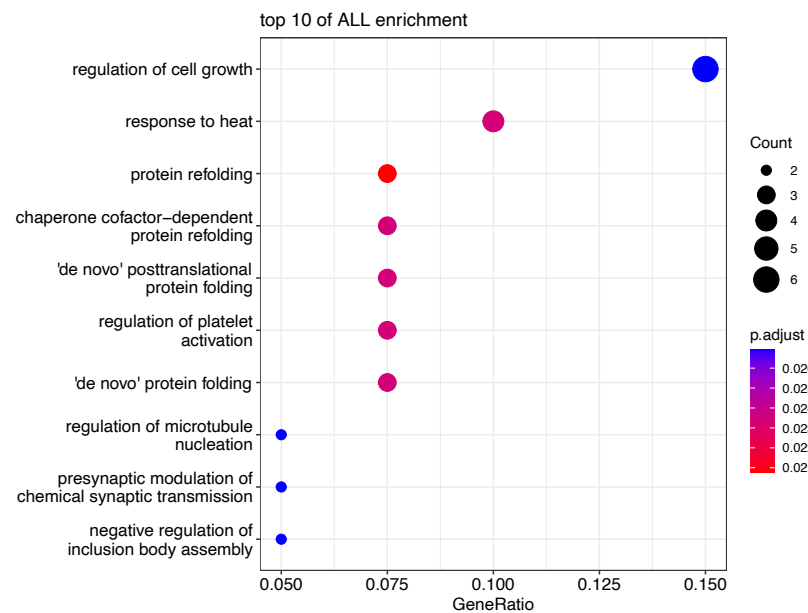

D

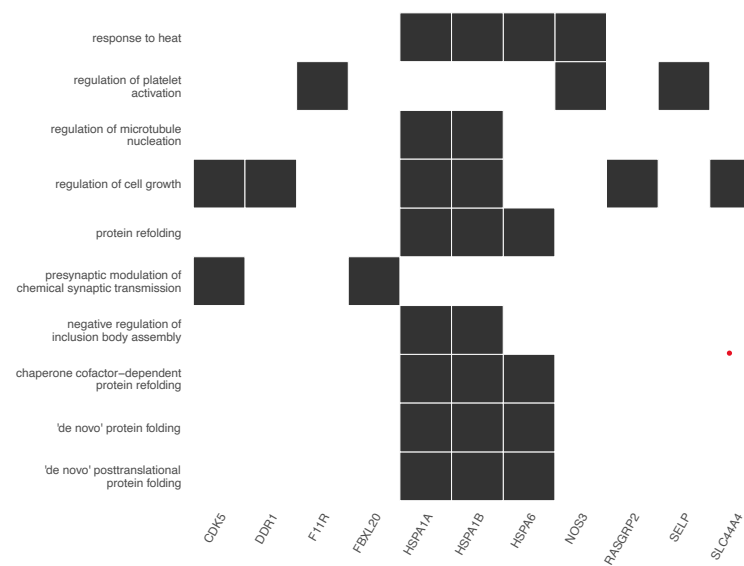

E

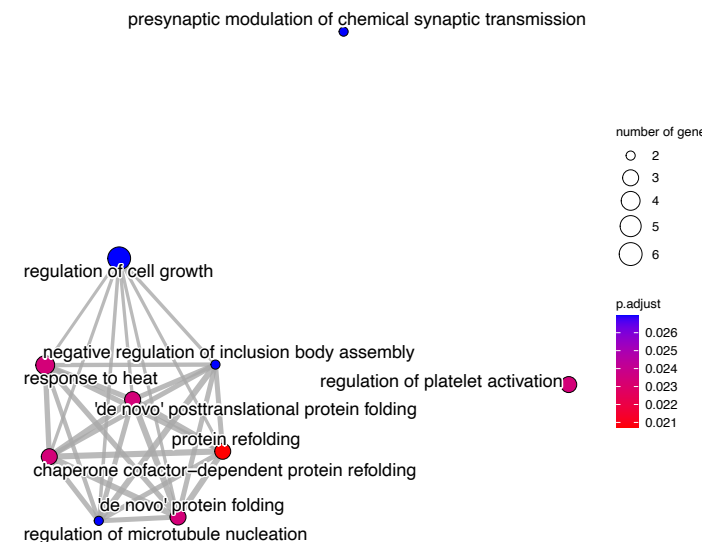

F

Supplement: Supplementary file 13 [file Image3.pdf]
